# Microgravity and immune cells

**DOI:** 10.1098/rsif.2022.0869

**Published:** 2023-02-15

**Authors:** Hongfang Lv, Huan Yang, Chunmei Jiang, Junling Shi, Ren-an Chen, Qingsheng Huang, Dongyan Shao

**Affiliations:** ^1^ Key Laboratory for Space Bioscience and Biotechnology, School of Life Sciences, Northwestern Polytechnical University, Xi'an 710072, People's Republic of China; ^2^ Hematology Department, Shaanxi Provincial Tumor Hospital, 309 Yanta West Road, Xi'an, Shaanxi 710072, People's Republic of China

**Keywords:** microgravity, differentiation, activation, metabolism, structure and function, recovery measures

## Abstract

The microgravity environment experienced during spaceflight severely impaired immune system, making astronauts vulnerable to various diseases that seriously threaten the health of astronauts. Immune cells are exceptionally sensitive to changes in gravity and the microgravity environment can affect multiple aspects of immune cells through different mechanisms. Previous reports have mainly summarized the role of microgravity in the classification of innate and adaptive immune cells, lacking an overall grasp of the laws that microgravity effects on immune cells at different stages of their entire developmental process, such as differentiation, activation, metabolism, as well as function, which are discussed and concluded in this review. The possible molecular mechanisms are also analysed to provide a clear understanding of the specific role of microgravity in the whole development process of immune cells. Furthermore, the existing methods by which to reverse the damage of immune cells caused by microgravity, such as the use of polysaccharides, flavonoids, other natural immune cell activators etc. to target cell proliferation, apoptosis and impaired function are summarized. This review will provide not only new directions and ideas for the study of immune cell function in the microgravity environment, but also an important theoretical basis for the development of immunosuppression prevention and treatment drugs for spaceflight.

## 1. Introduction

With the development of human science and technology, space flight has become a reality. The most important challenge that human beings face in the process of space exploration is determining how to adapt to the space environment, including microgravity, pressure, ionizing radiation and claustrophobia [1,2], all of which can cause certain damage to the human physiological state, such as an impaired immune system, bone loss, muscle atrophy, etc. [2]. Cogoli and Tschopp proposed, for the first time, that the change of gravity was the main cause of the damage to the human body caused by the space environment in the space mission carried out in 1983 [3]. Gravity plays an important role in regulating the dynamic homeostasis of tissues and cells, and it is the cells that undergo the most obvious changes when exposed to microgravity. Cells can convert the mechanical signal of changes in external gravity into intracellular biochemical signals, which results in the initiation of downstream signalling cascades [4], thereby affecting the shape, size and function of cells [5–7]. Ultimately, this manifests as changes in tissues, organs, systems or even whole biological individuals. These findings all indicate that changes in the gravitational environment can have important effects on the health of astronauts.

The human immune system has the functions of immune surveillance, defence and regulation, and plays an important role in recognizing and resisting pathogenic microorganisms; it coordinates with other systems of the body to maintain the stability and physiological homeostasis of the body's internal environment [8]. It has been reported that the immune system is one of the most severely affected systems during space flight [9,10], and an impaired immune system can pose a serious threat to the health of astronauts. Zhu *et al*. found that the retinoic-acid-inducible gene-I-like receptor (RLR) and Toll-like receptor (TLR) signalling pathways necessary for antiviral innate immunity were significantly inhibited in a microgravity environment, resulting in a dysfunctional antiviral immune response [11]. Bacterial or viral infections occurred in more than half of the astronauts on the Apollo mission, the first human mission to space in 1969. Conjunctivitis, upper respiratory tract infections, influenza, viral gastroenteritis, rhinitis and mild skin diseases have occurred during space flight [12]. Durnova *et al*. found the dysplasia of lymphoid organs (the spleen, lymph nodes and thymus) in rats that had undergone space flight [13]. Gridley *et al*. and Baqai *et al*. also found that spleen and thymus masses were reduced in post-flight mice [14,15]. Clinical data from 46 crew members on the International Space Station (ISS) showed that allergic reactions such as rhinitis and skin rashes were observed in many crew members [16]. The dysregulation of the immune system has been observed in both short-term and long-term space missions [9,17]. Sonnenfeld *et al*. and Zayzafoon *et al*. further confirmed that the changes in immune system function brought by space flight were mainly due to the microgravity environment [18,19]. Immune cells are abnormally sensitive to the microgravity environment [3], which affects their normal function and makes astronauts susceptible to various diseases. Therefore, increasingly more studies are now focusing on the effects of the microgravity environment on immune cells. Microgravity has been proven to inhibit the proliferation and activation of lymphocytes [20], affect the expression of cell surface molecules [21], and inhibit the movement and killing function of lymphocytes [22,23]. A decrease in monocytes has been observed in the blood samples of astronauts, the activation of macrophages has been found to be significantly inhibited [24], and the phagocytic and oxidative functions of neutrophils have been found to be significantly reduced [25]. The effects of microgravity on immune cells are summarized in table 1. These results suggest that alterations in gravity can affect multiple aspects of immune cells through different mechanisms, leading to impaired immune function and thus significantly increasing the health risk of astronauts. Therefore, understanding the effects of microgravity on immune cells and providing an in-depth analysis of the specific mechanisms involved are important prerequisites for establishing corresponding prevention and treatment measures and ensuring the health of astronauts.

**Table 1.** The effects of microgravity on immune cells. RCCS, rotating cell culture system; RPM, random positioning machine; RWV, rotating wall vessel.

cell types	cell source	cellular changes	microgravity means	references
macrophages	human	number of cells↓	flight, RCCS, ISS	[26]
		impaired polarization		[26]
		oxidative burst↓		[27]
		metabolic reprogramming		[26]
		cytoskeleton changes		[28]
DC	human mouse	number of cells↓	flight, RCCS	[29,30]
		number of cells↑		[31]
		phagocytosis↓		[32]
		antigen presentation↓		[33]
neutrophils	human	number of cells↑	flight	[24]
		morphological change	RCCS	[34]
		phagocytosis↓	parabolic flight	[25]
B lymphocytes	human mouse	number of cells↓	flight	[35]
		no change in number of cells	RCCS	[36]
T lymphocytes	human mouse	secreted cytokines↓	flight	[20,37]
		number of cells↓		[38,39]
		impaired activation	parabolic flight, RCCS	[40]
monocytes	human	oxidative burst↓	flight, ISS, RPM	[41,42]
		no change in number of cells		[37]
		migration ability↓		[37]
NK cells	human	number of cells↓	flight, ISS, RWV, RCCS	[38,43]
		kill capability↓		[23]
		no change in killing ability		[44]
eosinophils	mouse	number of cells↓	parabolic flight	[45]
basophils	mouse	number of cells↓	parabolic flight	[45]

However, to date, previous summaries and discussions provided in related studies have mainly focused on the effects of microgravity on two main classifications of immune cells, namely innate and adaptive immune cells. These studies lacked an overall grasp of the laws of the effects of microgravity on immune cells at different stages of their entire developmental process, such as their differentiation, metabolism and function. Therefore, this review mainly summarizes the effects of microgravity on immune cells and their related molecular mechanisms from the perspective of the immune cell development process, including their proliferation, differentiation, activation, metabolism and function. In addition, this review also summarizes the current means for restoring the function of immune cells damaged in the microgravity environment. Recovery of damaged immune cells in the microgravity environment is summarized in table 2. This provides an important reference for the development of drugs related to the prevention and treatment of damaged immune systems in astronauts, and provides theoretical guidance and new directions for future research on the effects of microgravity on immune cells.

**Table 2.** Recovery of damaged immune cells in the microgravity environment.

recovery means	recovery drug	recovery mechanism	reference
activation agent of signal pathway	NF-κB agonists IL-12, IL-15 and their combination	targeting the NF-κB signalling pathway to regulate the proliferation, activation, metabolism and apoptosis of immune cells	[26,38,46,47]
natural immune cell activator	mulberry leaf polysaccharide, polysaccharide extracted from *Cordyceps sinensis* mycelium	polysaccharides can regulate the proliferation and activation of immune cells by enhancing the expression of cytokines and surface receptors	[48]
	*N*-acetyl cysteine (NAC) and Quercetin O-Chipotle polysaccharide (GLP), *Lycium barbarum* polysaccharide (LBP) and *Lentinan* (LNT)	NAC and quercetin act as antioxidants and can inhibit the continuous phosphorylation of ERK, MKP-1 expression, and the caspase-3 activation of cells. Polysaccharides can also protect immune cells by inhibiting the apoptosis and necrosis induced by microgravity	[49]
	morin sulfates/glucuronides *Ganoderma lucidum* polysaccharide (GLP), *Lycium barbarum* polysaccharide (LBP) and *Lentinan* (LNT), etc.	the recovery of immune cell function by morin may be related to its activation or inhibition of protein phosphorylation. Polysaccharide may target TLR-4 and CR3 to restore immune cell function	[50,51]

## 2. The effects of microgravity on immune cells

### 2.1. The effects of microgravity on the proliferation and differentiation of immune cells

**Figure 1.**
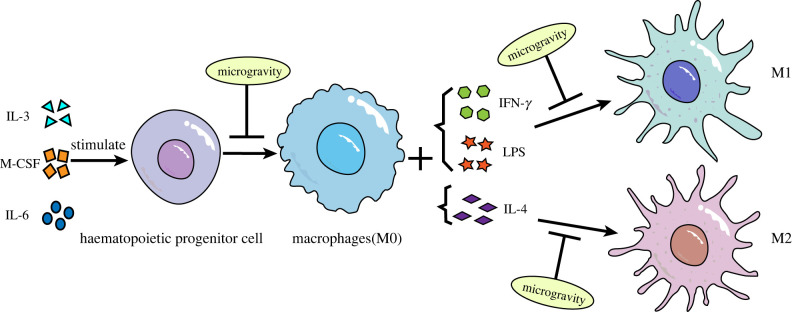
The effects of microgravity on macrophage differentiation.

Studies on the effect of microgravity on immune cell differentiation have mainly focused on macrophages. Macrophages are white blood cells located in tissues that are derived from the monocytes of haematopoietic stem cells. They participate in non-specific immunity *in vivo*, mainly in the form of fixed cells or free cells, to remove cell debris and pathogens. The monocyte-macrophage system is the body's first line of defence against viruses [52]. Studies have shown that microgravity significantly affected macrophage differentiation (figure 1). Shi *et al*. used macrophage colony-stimulating factor plus interleukin (IL)-3 and IL-6 to stimulate mouse bone marrow haematopoietic stem cells (HSCs) in space flight and a simulated microgravity (SMG) environment on the ground to observe the effect on the differentiation process. The results showed that the percentage and number of macrophages were significantly reduced, and the rate of cell proliferation was also significantly slowed down [26]. These results suggest that microgravity significantly inhibits the differentiation of HSCs into macrophages. Racine *et al*. also found that the percentage of monocytes transformed into macrophages and the number of macrophages was significantly reduced under microgravity environment, suggesting that the differentiation process from monocytes to macrophages is also significantly affected by microgravity [53]. Previously, RNA-seq was used to analyse the transcriptome of macrophages differentiated by space and ground control for 12 days. The gene ontology (GO) analysis and Kyoto Encyclopedia of Genes and Genomes (KEGG) results showed that five major signalling pathways related to cell proliferation and differentiation (RAS, ERK, NF-κB, etc.) were significantly downregulated, which further indicates that microgravity affects the differentiation process of macrophages [26]. In addition, macrophages can be classified into three phenotypes: the non-polarized M0 type, the pro-inflammatory M1 type and the anti-inflammatory M2 type. In previous research, interferon-gamma (IFN-γ) and lipopolysaccharide (LPS) were used to stimulate the M1-type polarization of macrophages, and IL-4 was used to stimulate the M2-type polarization of macrophages [54,55]. By detecting the expression levels of M1 (tumour necrosis factor *α* (TNF-α), I-Ab) and M2 (Arg-1, CD206) macrophage marker molecules, it was found that the responses of macrophages to IFN-γ and LPS or IL-4 under microgravity conditions were less sensitive than those of macrophages under normal gravity conditions, suggesting that the polarization process of macrophages to M1 and M2 was impaired in the microgravity environment [26]. Because M1- and M2-type polarization play an important role in the immune function of macrophages, the influence of microgravity on their differentiation process may be an important reason for their impaired immune function.

**Figure 2.**
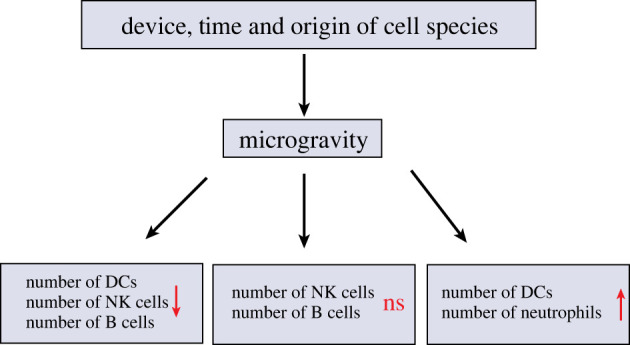
The effects of microgravity on the number of immune cells.

Dendritic cells (DCs) are an important component of the innate immune system and are the most powerful antigen-presenting cells in the body. They can recognize and present antigens and activate T cells, which are mainly differentiated from haematopoietic stem cells. Microgravity can have an effect on the number of DCs (figure 2); Savary *et al*. found a significant decrease in DC production after 9–12 days of SMG conditions using the rotating cell culture system [32]. Studies have also shown that the differentiation of haematopoietic stem cells into DCs in astronauts who had experienced space travel for 10 days was significantly impaired, resulting in a decrease in the number of DCs [29,30]. However, contrary to these results, Chen *et al*. found a significant increase in the number of DCs after treating splenocytes with a rotary bioreactor system to simulate microgravity for 16 h [31]. It is speculated that the difference in the results may be due to the different methods of microgravity generation and varying durations. However, there have been fewer studies on the specific mechanism by which microgravity affects the DC differentiation process, and the exact cause still requires further exploration.

Neutrophils are immune cells derived from bone marrow. They are the first to reach the site of infection and have chemotaxis, phagocytosis and bactericidal effects. They play an important role in the prevention of infection and are an important factor affecting the health of astronauts [56]. To date, there have not been many studies on the effects of microgravity on neutrophils, and related research has mainly focused on two aspects: quantity and function (figure 2). The number and proportion of neutrophils were also found to be increased in blood samples from 25 astronauts during four space missions [24]. Stowe *et al.* also reported an increase in neutrophil numbers and altered function after space flight [57]. Stervbo *et al*. found that the number of granulocytes increased during parabolic flight, with a significant increase in the number of neutrophils and a significant decrease in the numbers of basophils and eosinophils [45]. Kim *et al*. reported that mast cells (BMMCs) showing reduced proliferation and increased apoptosis, as well as impaired degranulation and secretion of IL-6,TNF-α in a microgravity environment [58]. Kaufmann *et al*. found that parabolic flight induced an increase in the number of leucocytes and significantly increased the percentage of neutrophils [59]. Moreover, the ratio of neutrophils to lymphocytes could be used as an indicator of health testing for space flight personnel, with important implications for predicting the development of inflammation and cancer [60]. However, the exact mechanism of the increase in the proportion and number of neutrophils is unknown, and it is speculated that it may be due to the promotion of neutrophil maturation by microgravity, the release of more neutrophils from the bone marrow and the reduction of neutrophil apoptosis [25,41]. However, Wang *et al*. used a RCCS to simulate microgravity, and did not find clear evidence of neutrophil maturation. Moreover, the rate of apoptosis remained unchanged under RCCS exposure as compared with 1*g*. Thus, it is speculated that the increased number of neutrophils may be due to the release of more neutrophils from the bone marrow. Furthermore, it was also found that the neutrophil model HL-60 cells secreted increased levels of IL-8, which could induce the release of more neutrophils from the bone marrow. Therefore, the increased secretion of IL-8 may also be a reason for the increase in the number of neutrophils [34,61,62] to supply the impairment of innate immune function in the microgravity environment [16]. However, at present, the mechanism by which microgravity increases the number of neutrophils can only be speculated, and the exact mechanism requires further study.

Natural killer (NK) cells are derived from bone marrow lymphoid stem cells, and their differentiation and development depend on the bone marrow and thymus microenvironment. Different from T and B cells, NK cells can recognize and kill tumour cells without pre-sensitization, and are important effector cells of the innate immune system for the immune surveillance of tumours and viruses [63,64]. At present, the results of studies on the changes of NK cell numbers in the microgravity environment are inconsistent (figure 2). A study using a rotary bioreactor system to simulate the microgravity environment for 16 h found that the number of NK cells in mice was not significantly affected [31]. However, in some studies, a two-dimensional rotating wall vessel (2D-RWV) was used to simulate the microgravity environment for 24 and 48 h, and it was found that microgravity could inhibit the proliferation of human NK cells induced by IL-2 and promote the apoptosis of NK cells, thereby reducing the number of NK cells [65]. The authors' previous study also found that NK cell survival was significantly reduced after 24 and 48 h of SMG treatment with a 2D-RWV [43]. Li *et al*. also found that microgravity treatment led to the increase of apoptosis and necrosis, resulting in a decrease in the number of NK cells [38]. Although there is controversy surrounding the effect of microgravity on the number of NK cells, most studies have found that microgravity treatment can affect the proliferation and apoptosis of NK cells, resulting in a decrease in the number of NK cells.

B lymphocytes are differentiated from bone marrow pluripotent stem cells. In peripheral blood, B cells account for approximately 10–15% of the total number of lymphocytes and are the only immune cells in the body that can produce antibodies, thus becoming an important component of humoral immunity. B cells have antigen recognition receptors on their surface that can recognize conformation-determining clusters of soluble protein antigen molecules, which allow B cells to activate, proliferate and differentiate into plasma cells that can synthesize and secrete various immunoglobulins [66]. Post-space-flight data show that the microgravity environment affects the humoral immune response of amphibians in terms of the number of immune cells, and it was also found that the number of B cells in the spleen of mice flying on biosatellites for one month was decreased by 41%, thus demonstrating that the microgravity environment adversely affected the number of B lymphocytes in mice (figure 2) [35]. However, Spielmann *et al*. found that the number of B-cell subpopulation numbers and the contents of IgG and IgM did not change significantly after a six-month mission by the ISS crew, suggesting that the homeostasis of B cells was maintained during the space flight [36]. These differences in results may have been due to the duration of B cells in microgravity and the different species origins of B cells. Furthermore, some studies have used an RWV bioreactor to treat HMy2.CIR cells, and found that SMG increased the cell apoptosis induced by heavy ion radiation. Further studies found that microgravity can promote the generation of intracellular reactive oxygen species (ROS), thus inducing the activation of the ROS-sensitive ERK/MKP-1/caspase-3 pathway, which may be the main reason for the increased apoptosis and decreased number of B lymphocytes [49]. However, most studies have focused on the effects of space flight and SMG on innate immune cells. There have not been many reports on humoral immunity and B lymphocytes, which may be because antibodies and other substances related to humoral immunity are difficult to collect and observe under microgravity conditions. In the future, more methods will need to be discovered to study the effects of microgravity on B cells.

### 2.2. The effects of microgravity on immune cell activation

#### 2.2.1. Dendritic cells

**Figure 3.**
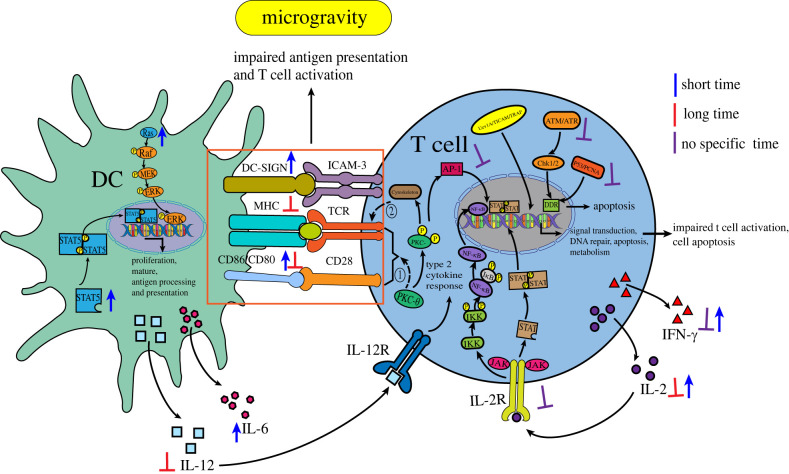
The effects of microgravity on immune cell activation. DDR, DNA damage response; Uev1A/TICAM/TRAF, anti-apoptosis pathway; ① PKC-θ participates in the integration of TCR and costimulatory molecule CD28; ② the destruction of T lymphocyte skeleton will lead to the damage of TCR activation.

Most of the DCs in the body are in an immature state and have a strong ability to phagocytose antigens. When they encounter foreign microorganisms, they will upregulate the expression of surface proteins (MHC class I and II molecules (signal 1), T cells co-stimulatory ligands, CD80, CD86 and 4-1BBL (signal 2)) and secrete cytokines (signal 3) to become mature DCs. At the same time, these three signals are necessary to stimulate the proliferation and differentiation of T cells, and then activate T cells [67]. Savary *et al*. used an RCCS to simulate microgravity treatment for 9–12 days, and found that the ability of DCs to phagocytose *Aspergillus fumigatus* Conidia in the microgravity environment was not as good as that of the ground control group. When faced with fungal antigens under the microgravity environment, the level of IL-12 production was lower, and the downregulation of IL-12 further affected the partial downregulation of some stimulatory molecules related to antigen presentation. Moreover, the molecules HLA-DR (MHC class II molecule) and CD56, which are essential for the activation of T lymphocytes on their surface, were also downregulated [32]. This result indicates that the antigen-presenting ability of DCs towards pathogens was inhibited by microgravity. However, in another study, it has also found that within 72 h of RCCS SMG treatment, microgravity induced the activation of the STAT5 signalling pathway, which is essential for DC proliferation and the ability to stimulate T cells (immunogenicity), and simultaneously induced the activation of the MAPK signalling pathway, which promotes DC maturation and antigen processing and presentation. Moreover, the DCs expressed more mature DC-SIGN markers (DC-SIGN contributes to T cell activation costimulation (signal 2)), CD86, and CD80, released more IL-6, and promoted T cells to produce more IL-2 and IFN-γ; this indicates that short-term SMG treatment better promoted DC maturation and the activation of T cells as compared with the normal gravity group [68]. In addition, Chen *et al*. also found an increase in the number of DCs in the immune cell subsets of mice treated with the rotary bioreactor system to simulate a microgravity environment for 16 h [31], which also proved that transient microgravity treatment was beneficial to the development of DCs. The difference in these results may have been due to the difference in the time spent in the microgravity environment; overall, with the increase of the SMG treatment time, DCs will be damaged to some extent, such as via the change in the dendritic distribution and the low expression of CD40, CD80 and CD86, which will lead to the damage of antigen uptake and T cell activation [33]. The diminished antigen activation of T cells via the inhibition of the secretion of IL-2 in the T cells can further reduce the antigen responsiveness of T cells. The reduction of IL-2 also inhibits the production of regulatory T cells, which puts the body at risk of autoimmune diseases or inflammation [68]. These findings suggest that while short-term microgravity treatment promotes the development of DCs, they may suffer some damage when exposed to microgravity for a long period of time, which significantly inhibits their ability to activate T cells. See figure 3 for relevant content.

#### 2.2.2. T cells

T lymphocytes are lymphatic stem cells derived from the thymus gland, and after differentiation and maturation in the thymus gland, they are distributed to immune organs and tissues throughout the body through lymphatic and blood circulation to perform immune functions. They are one of the most important adaptive immune cells that play an important role in the process of cellular immunity and the maintenance of body homeostasis. To date, there have been many related studies on the effect of microgravity on the activation process of T cells. Studies conducted in several space laboratories have revealed a significant loss of human T cell activity in peripheral blood leucocytes during flight, as well as time-dependent decreases in proliferation, expressions of surface activation-type markers CD25, CD69 and CD71, and secretions of inflammatory cytokines in CD4+ and CD8+ T cells exposed to microgravity as compared with T cells exposed to normal gravity. Additionally, the inhibition of the activation response to their receptor activator knife-bean globulin A (ConA) has been found to reach 97% [20,37]. By using RWV, Spatz *et al*. also found that microgravity simultaneously inhibited the ability of CD4 + T and CD8 + T to respond to a robust activation stimulus (decreased CD25, CD69 and JAK/STAT responses), while enhancing immunosuppressive Treg responses (increased STAT5 related responses) [69]. Moreover, in terms of proliferation, CD4+ T cells have been found to be more sensitive to microgravity than CD8+ T cells [70]. In addition, the number of T lymphocytes was found to be decreased [38,39] and their activity was inhibited in both real and SMG experiments in rats [71]. These results suggest that microgravity negatively affects T cells.

Further analysis of the mechanisms by which microgravity affects T cells has revealed that in ground-based SMG and parabolic flight, the secretion of IL-2 and IFN-γ by human and animal T cells in the microgravity environment was correspondingly reduced [33]. Similarly, Martinez *et al*. found that key genes such as IL-2 and IFN-γ were significantly downregulated under both real microgravity and SMG conditions [72]. As a growth factor of T cells, not only can IL-2 stimulate the long-term continuous proliferation of T cells [73], but, as an activation pathway of T cells, IL-2 and IL-2R can only trigger the complete activation of T lymphocytes when they interact with each other. Some studies have shown that the expressions of IL-2 and IL-2R were significantly downregulated under the microgravity environment [74], which suggests that the downregulation of IL-2 and IL-2R may be one of the reasons for the decrease in the number and activity of T cells due to microgravity [40]. Boonyaratanakornkit *et al.* further found that the IL-2R downstream signaling pathways NF-κB and JAK-STAT were downregulated under microgravity conditions [75]. Furthermore, promoter region analysis has revealed that the impairment of NF-κB, AP-1 and STAT may be responsible for the significant downregulation of the expression of many genes involved in signal transduction, DNA repair, apoptosis and multiple metabolic pathways, including pro-inflammatory cytokines and chemokines, under microgravity conditions [75,76], which leads to impaired activation of T cells [77]. In addition, Zhao *et al*. used an RWV to simulate microgravity, and, via microarray analysis, observed the deregulation of 79 genes containing signal transduction, DNA repair, apoptosis and multiple metabolic pathways; they suggested that SMG promotes the apoptotic response by inhibiting the anti-apoptotic pathways regulated by Uev1A/TICAM/TRAF/NF-κB and the DNA damage response pathways controlled by p53/PCNA and ATM/ATR-Chk1/2 [78]. These results suggest that microgravity may affect the expression of IL-2 and its receptors, which affect various signalling pathways, including cell proliferation, differentiation, apoptosis, DNA repair and other metabolic pathways, and subsequently affect the number and activation process of T cells, thus leading to impaired T cell immune function. See figure 3 for relevant content.

Regarding the mechanism of impaired T cell activity in the microgravity environment in addition to the previously mentioned causes [79], alterations in protein kinase C (PKC) translocation and activation may be another important cause (figure 3) [80]. PKC is a member of the serine/threonine kinase family and plays a key role in the signal transduction of eukaryotic cell proliferation and differentiation [81]. PKC*θ*, a member of the novel PKC subtype in the PKC family, is the only PKC subtype that translocates to the immune synapse after the antigen stimulation of T cells. It is able to participate in the integration of T cell receptor (TCR) and co-stimulatory molecule CD28 signals, and these synergistic signals are important for T cell activation. PKC*θ* is also an important factor in the activation pathway of activation protein AP-1 induction, and is also an essential component in immune synapses; thus, PKC is necessary for T cell activation [82,83]. It has been shown that microgravity impaired the translocation of PKC and the number of PKC particles, thus impairing the signalling processes associated with these kinases [80]. Hatton *et al*. also found that the amount of PKC in activated human peripheral blood Jurkat T cells was significantly decreased in the microgravity environment [84]. Schmitt *et al*. used radioisotope-labelled PKC activator phorbol ester (3H-PDBu) to detect the relative distribution of PKC in the cytoplasm and nucleus with the increase of *g*; it was found that cytoplasmic PKC increased and nuclear PKC decreased, but the total amount of PKC in the cytoplasm and nucleus remained constant [85]. These results suggest that microgravity can affect the function of T cells by affecting the number and migration of PKC. It is important to note that the translocation of PKC is closely associated with the change of the cytoskeleton [86]. Changes in the organization of vimentin filaments and a decrease in mitogens have been observed in Jurkat cells experiencing microgravity during space flight, which can have an impact on the cytoskeleton of Jurkat cells [87]. Lewis *et al*. used DNA microarray analysis to find changes in some genes related to the cytoskeleton in Jurkat cells exposed to microgravity for 48 h [88,89]. Bradley *et al*. found that the disruption of the T lymphocytes cytoskeleton can lead to the impaired activation of the TCR, thus allowing cells to be immunosuppressed under microgravity [90,91]. Therefore, based on the fact that changes in the T lymphocytes cytoskeleton under the microgravity environment are closely related to the translocation of PKC, it is speculated that the changes of PKC may be another important reason for the impaired proliferation and activation of T lymphocytes. At present, the mechanism by which changes in PKC interact with the cytoskeleton to affect T cell function remains unclear, which can be used as an entry point for further research in the future.

### 2.3. The effects of microgravity on immune cell metabolism

**Figure 4.**
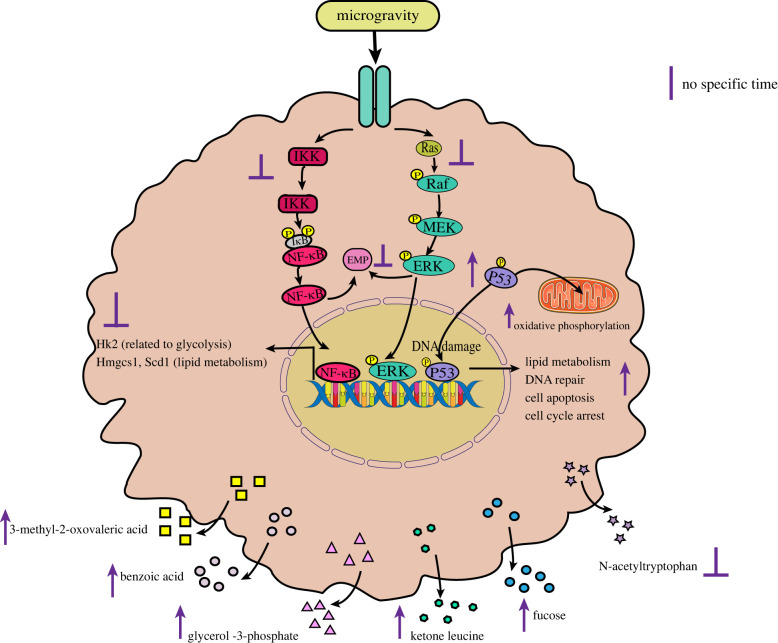
The effects of microgravity on immune cell metabolism.

On the whole, there have been few studies on the effects of microgravity on immune cell metabolism, and related research is mainly focused on macrophages. Transcriptome analysis via the RNA-seq of macrophages differentiated in space and ground control for 12 days, GO analysis, and KEGG results have revealed that five major signalling pathways (RAS, ERK, NF-κB, etc.) for cell proliferation and differentiation were significantly downregulated, and the pathways of metabolic processes such as lipid metabolism and nucleotide metabolism were also altered to varying degrees [26]. These results indicate that microgravity affects not only macrophage differentiation, but also a series of metabolic pathways in macrophages. Tauber *et al*. found significant changes in the abundance of several metabolites in the supernatant of human primary macrophages cultured for 11 days on the ISS [92]. Compared with those under the ground control conditions, the contents of 3-methyl-2-oxopentanoic acid (a marker of metabolic disorders of branched amino acids), benzoic acid, glycerol-3-phosphate (the reduction of intracellular NADH to ATP, and the de novo synthesis of glycerides) [93], ketoleucine (4-methyl-2-pentanoic acid) and fucose were found to be significantly increased, and the content of N-acetyltryptophan was found to be significantly decreased [92,94]. However, the decrease of N-acetyltryptophan may suggest a decreased ability of cells to produce nitric oxide [95], which would lead to a decrease in the defence of macrophages against microbial infection. Shi *et al*. conducted a study of real and ground-based SMG environments, and found that the lack of macrophage production and impaired function may also be achieved through RAS, ERK, NF-κB and p53, as well as other signalling pathways. Under the influence of these pathways, the key metabolic genes and proteins related to glycolysis, such as Hk2, and to lipid metabolism, such as Hmgcs1 and Scd1, were further downregulated [26], which further affected the immune function of macrophages (figure 4). These studies have shown that macrophage metabolism is reprogrammed under microgravity conditions, which will lead to the damage of some metabolic pathways and ultimately affect macrophage function. However, there has been no report on the specific mechanism by which microgravity affects macrophage metabolic pathways; this research would allow the relevant mechanism to be studied in depth, thus providing an important theoretical basis for the screening and development of relevant targeted drugs.

### 2.4. The effects of microgravity on the structure and function of immune cells

#### 2.4.1. Monocytes

**Figure 5.**
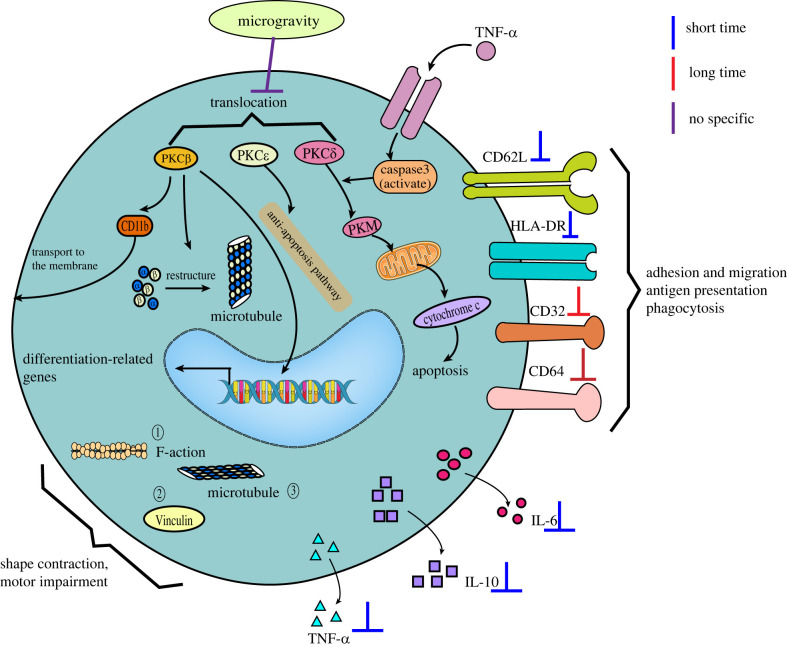
The effects of microgravity on the structure and function of monocytes. ① The density of filamentous polymers of F-actin was found to be significantly reduced, and the cytosolic network disappeared, mainly distributed near the plasma membrane; ② Vinculin lost its radial orientation and aligned parallel to the cell membrane; ③ the microtubules, responsible for cell division, did not display their typical radial array; they were highly disorganized, and showed a more evident thickening in perinuclear position and a surrounding arborization.

Monocytes, as the largest blood cells in the blood, are also the largest leucocytes, which are derived from haematopoietic stem cells in the bone marrow and developed in the bone marrow. They are currently considered to be the precursors of macrophages and DCs. Monocytes are an important component of the body's defence system and are capable of shape-shifting movement. In addition to being able to phagocytose foreign objects, monocytes can also migrate to the site of inflammation under local inflammation and participate in local immune responses; thus, they play an important role in the process of phagocytosis and the removal of injured and senescent cells and their debris. Monocytes can also transfer their antigen determinants to lymphocytes after the phagocytosis of antigens to participate in immune response [96]. Therefore, recent studies on the effects of microgravity on monocytes have focused on the expression of surface molecules and the alteration of intracellular structures associated with their phagocytosis and migratory movements (figure 5).

Crucian *et al*. collected peripheral blood mononuclear cells from nine astronauts who experienced a short-duration space flight. The results showed that although the cell numbers did not change significantly, the surface expressions of CD62L and HLA-DR, key factors associated with endothelial cell adhesion and tissue migration, were significantly reduced [37]; this would lead to the significant inhibition of monocyte adhesion and tissue migration motility, as well as the significant impairment of antigen presentation, resulting in the impaired immune function of monocytes. A study by Kaur *et al*. similarly found that the expressions of two surface markers associated with phagocytosis (CD32 and CD64) on monocytes were decreased in astronauts who underwent 5–11 days of space flight. It was also found that the abilities of monocytes to phagocytose *Escherichia coli*, trigger oxidative burst, degranulation, and produce cytokines after stimulation all decreased [41,42].

Meloni *et al*. found via ISS experiments that microgravity affects the skeletal structure of monocytes and their ability to migrate [97]. Compared with the ground control group, the density of filamentous polymers of F-actin exposed to microgravity was found to be significantly reduced, and the cytosolic network disappeared, mainly distributed near the plasma membrane. Moreover, the structure of β-microtubulin was found to be disrupted, the microtubules responsible for cell division were highly disorganized, and obvious thickening and surrounding dendritic structures were found in the perinuclear position. Vinculin was found to lose its radial orientation and was aligned parallel to the cell membrane. Changes in the content and distribution of these protein fibres were found to significantly shrink the cell morphology of J111 monocytes and seriously impair their motor ability [97]. Furthermore, random positioning machines were used to simulate microgravity treatment for 1 h; it was found that the cytoskeleton structure of J-111 cells changed significantly, the density of filamentous polymers in the F-actin network decreased significantly, and actin stress fibres presented local continuous subplasmic bundles [80]. All these findings indicate that the microgravity environment can lead to significant changes in the cytoskeleton of monocytes, which may significantly affect their movement ability, further affect the migration of monocytes to the inflammatory site, and affect the local immune response to a certain extent. These studies indicate that the structure and function of monocytes are gravity-sensitive.

In addition, similar to T cells, microgravity has been shown to affect the function of monocytes by inhibiting the PKC signalling pathway [80]. The activation of PKC can not only regulate a series of biochemical reactions involving the PKC in cells, but can also act on transcription factors in the nucleus to regulate gene expression and play a key role in the signal transduction process of eukaryotic cell proliferation, differentiation and apoptosis regulation [98]. Different PKC isoforms play different roles in regulating monocyte differentiation and apoptosis. PKC*β*II is involved in the regulation of microtubule reorganization, CD11b trafficking to the cell surface (necessary for cell adhesion), and the expression of early genes required for differential expression. PKC*ε* is involved in the anti-apoptotic process of cells. PKC*δ* plays a role in the initiation response of apoptosis. When apoptosis-inducing agents such as TNF-α activate caspase 3, PKC*δ* is then cut into active catalytic fragments (PKM) and translocated to the mitochondria, which mediates the release of cytochrome C and induces apoptosis. The translocation of PKC subunits typically occurs during PKC activation. Studies have shown that microgravity can reduce the translocation of various subtypes of PKC in human mononuclear cell line U937 and T cells to varying degrees, thereby impairing the signal transduction process related to these kinases, which may then affect the related processes of cell activation, differentiation, apoptosis and the cell cycle [80,84]. In addition, PKC signalling also plays a certain role in the movement of cells [86], so it is speculated that changes in the PKC signalling pathway may also be one of the important reasons why microgravity reduces the motility of monocytes and thus affects their function. However, at present, there is no clear evidence of whether microgravity affects monocyte motility by affecting the PKC signalling pathway. The mechanism of the microgravity-induced impairment of monocyte migration can be further explored from this perspective. In addition, some studies have found that the expression of various surface marker molecules (CD62L, HLA-DR, CD11a, etc.) and the secretion of inflammatory factors (TNF-α, IL-6 and IL-10) of monocytes were inhibited after short-term space flight, which may be another important reason for the impairment of monocyte function [37].

#### 2.4.2. Macrophages

**Figure 6.**
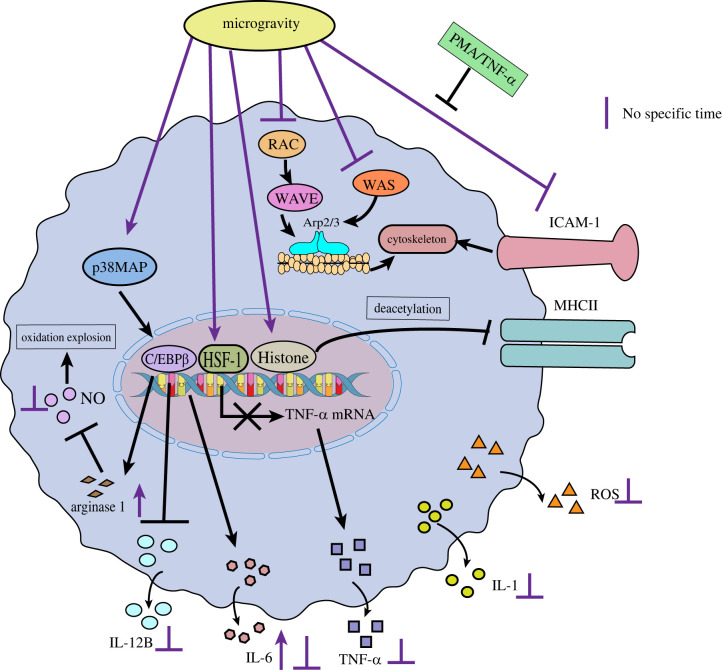
The effects of microgravity on the structure and function of macrophages.

The effects of SMG on the structure and function of macrophages and their related mechanisms are the most extensively investigated. Tauber *et al*. found that the immune dysfunction in the SMG environment may be the result of the regulation of the cell adhesion molecule ICAM-1 in the monocyte/macrophage system, and ICAM-1 is considered as a possible molecule for rapid response and continuous gravity regulation in mammalian cells (figure 6) [92]. The decreased expression of ICAM-1 in macrophages and the absence of cell surface binding sites may lead to the impairment of the T cell activation, migration and activation of innate immune responses [92]. This is because the regulation of ICAM-1 expression is associated with the function of the cytoskeleton, which acts as a scaffolding system for cells and plays an important role in maintaining cell morphology, cell migration, antigen recognition, intracellular transport, signal transduction and the phagocytosis of macrophages, and is an important structural basis for the ability of macrophages to deform and migrate [28]. Changes in ICAM-1 expression directly lead to alterations in the cytoskeleton of macrophages [99], which leads to an impact on skeleton-related functions. Current studies have shown that changes in the protein expression level of ICAM-1 are not consistent under microgravity. The expression of ICAM-1 is increased in human U937 macrophages, while the expression of ICAM-1 is significantly decreased in mouse BV-2 cells [100]. The differences between the ICAM-1 regulation of macrophage-like differentiated human U937 and murine BV-2 microglial cells in microgravity could be the result of the different species (murine and human) or different molecular and functional features of peripheral macrophages and central nervous system (CNS) macrophages. Interestingly, the mRNA expression level of ICAM-1 has been found to remain largely unchanged in these cells [101]. It is speculated that microgravity may only affect the protein expression of ICAM-1 by affecting its translation process, and has no significant effect at the transcriptional level. Another study found that the use of phorbol-12-myristate-13-acetate (PMA) or an appropriate concentration of the pro-inflammatory factor TNF-α would weaken or even offset the inhibitory effect of microgravity on ICAM-1 expression, but when a high concentration of TNF-α was treated, the result was apoptosis [100]. This provides ideas for the development of relevant drugs, but also prompts the consideration of the effects of the drug dosage. In another study, magnetic levitation was used to simulate microgravity to treat mouse RAW264.7 cells. It was found that microgravity could decrease the expression of RAC1, WASP2, WAVE2, ARP2 and other genes in the RAC-WAVE-Arp2/3 composite signalling pathway related to cytoskeleton synthesis, resulting in a decrease in the synthesis of the cytoskeleton; this not only affects the morphology of the cell, but also further affects the phagocytosis of the cell [102]. While there is no relevant report on whether there is a correlation between the regulation of ICAM-1 expression by microgravity and changes in the RAC-WAVE-Arp2/3 signalling pathway, in the future, the mechanism by which microgravity affects macrophage cytoskeleton changes can be studied in depth from this perspective. In addition, major histocompatibility complex class II (MHCII) molecules are important antigen-presenting molecules, and microgravity also downregulates MHCII molecules on the surfaces of macrophages by deacetylating histones, thereby affecting specific immune responses [99].

Under microgravity conditions, macrophages also exhibit disturbed cytokine release [41,103] and reduced oxidative burst (figure 6) [27]. For example, data from the ISS Laboratory have shown that, at the protein level, the levels of IL-1, IL-6, TNF-α and ROS produced by macrophages were significantly reduced after space travel [14,104]. Wang *et al*. also found that the expression of arginase I in macrophages was significantly upregulated and the protein expression level of TNF-α was downregulated under SMG (RCCS) conditions, but SMG did not affect the stability of TNF-α mRNA [101,104]. Related molecular mechanism studies revealed no significant changes in TLR4 expression and NF-κB nuclear translocation (which regulates the transcriptional signalling pathway of multiple inflammatory cytokines including TNF-α) in LPS-induced macrophages under SMG conditions. This suggests that microgravity would not affect the TLR4/NF-κB signalling pathway, and thus would not affect the transcriptional process of TNF-α through this pathway; however, heat shock factor-1 (HSF1) was found to be significantly activated as an inhibitor of the TNF-α promoter, resulting in the reduced expression of TNF-α mRNA [104]. In addition, Wang *et al*. examined the intracellular expression of related signals and molecules, and found that microgravity upregulated the expression of macrophage arginase promoter transcription factor C/EBP*β* by activating the p38 MAPK signalling pathway, which upregulated macrophage arginase1 and IL-6 expression and downregulated IL-12B expression [105]. However, the upregulation of arginase1 expression and the activation of its associated signalling pathways lead to decreased NO production, which can affect the oxidative burst capacity of macrophages. Furthermore, it was found that the transcriptional activator STAT6 was not involved in the upregulation of arginase1 expression caused by microgravity [105]. Collectively, these changes may disrupt the balance between pro- and anti-inflammatory systems, thus impairing macrophage immune responses [101]. It has also been reported that the effect of microgravity on macrophages is limited, and that the inhibitory effect of microgravity on macrophages lasts only for a short period (less than 1 min) [106]. Under the condition of SMG, the expression of TLR4 (lipopolysaccharide receptor) and its downstream signalling pathways are restored in a very short period (a few seconds), thereby also allowing the oxidative burst capacity of macrophages to be restored in a very short period [25], which suggests that macrophages have the ability to adapt to the microgravity environment. In conclusion, at this stage, the effects of microgravity on macrophages and their specific molecular mechanisms are not fully understood. However, macrophages play a key role in the process of antigen presentation [107], and the effect of the microgravity environment on macrophages will limit the extent of the organism's response to bacteria or fungi, leading to a failure in the control of pathogenic bacteria [108], which may have a great impact on the whole immune system. Therefore, more research is needed to fully elucidate the molecular mechanisms by which microgravity interferes with macrophage physiology, and thus to develop effective measures to counteract these adverse effects. In addition, according to the existing mechanism, inhibitors of HSF1 and the signalling pathway of arginase1 expression can be screened, and drugs to restore the function of macrophages can be developed, thereby alleviating the impact of microgravity on the health of space mission personnel.

#### 2.4.3. Neutrophils

**Figure 7.**
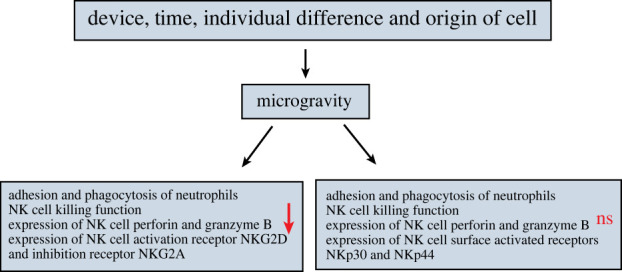
The effects of microgravity on the structure and function of neutrophils and NK cells.

It has been found that after short-term (8–15 days) space flight, the capacity for the adhesion between neutrophils and endothelial cells is significantly increased in astronauts, which enhances the neutrophil response to inflammatory mediators [57]. Wang *et al*. used an RCCS to simulate microgravity and found some changes in the neutrophil (PMN)-like HL-60 cell morphology and a significant increase in cell adhesion molecule expression [34]. However, another study found that the adhesion and phagocytic properties of neutrophils did not change significantly after 30 parabolic flight manoeuvres in 21 healthy male volunteers [59]. It is speculated that the reason for the different results may be due to the influence of different microgravity handling methods and individual differences in experiments or different cell sources. Furthermore, during a 5- to 11-day flight mission, the phagocytosis and oxidative burst capacity of neutrophils against *E. coli* were not significantly different from those in normal gravity when observed after 5 days of the mission. However, their phagocytosis and oxidative burst capacity were found to be significantly lower than those under normal gravity before and after a 9- to 11-day mission. This suggests that neutrophil phagocytosis and oxidative function may be influenced by the flight duration [25]. Combined with previous reports that indicate that the space flight duration may have a crucial impact on neutrophil function, it is speculated that as the number of neutrophils is increased during brief exposure to microgravity and the expression of cell adhesion molecules is upregulated [109], the host defence function is enhanced to complement the impairment of immune function; however, with the increase of the exposure time, neutrophil phagocytosis and oxidative functions may be inhibited to some extent (figure 7). However, it is not clear for how long microgravity exposure will stimulate neutrophils and for how long it will inhibit the function of neutrophils, and the relevant mechanisms must be further studied. In addition, after space flight, changes in the levels of hormones such as epinephrine have also been found along with changes in neutrophil adhesion [57]. A study in which endothelial cells were exposed to epinephrine or epinephrine was directly injected into the serum of subjects found decreased neutrophil adhesion [110]. Therefore, it is speculated that the changes of neutrophil adhesion caused by microgravity may be related to various stress hormones in the body. However, it is not clear which specific hormones will have an effect, and the relevant mechanisms require further investigation.

#### 2.4.4. Natural killer cells

To date, the research on NK cells has focused on the effects and mechanisms of microgravity on their killing function. Bigley *et al*. analysed crew samples collected during the flight phase on the ISS, and found that space microgravity exposure resulted in a significant reduction in the killing capacity of NK cells [23]. Additionally, on-board flight experiments conducted on the Salyut 6 and 7 and Mir orbital stations revealed a significant decrease in the killing activity of NK cells in astronauts within a week after a long space flight [111], and it was found that astronauts experiencing microgravity for the first time were more severely damaged [23]. It is speculated that there is an adaptive regulation of the response of NK cells or the human immune system to microgravity. It has also been found that after 9–10 days of space flight, the killing activity of NK cells in the body was decreased by 40% as compared with that before the flight, and the activity of only 3 out of 10 people returned to the pre-flight level 3 days after landing [112]. The NK cell activity of astronauts who have experienced 8–366 days of space flight (or space station life) has been compared before and after flight, and it was also found that the NK cell killing activity was significantly reduced; it took half a month for most astronauts to recover to the normal level of cell activity, and some even took two months [113]. Using 2D-RWV to simulate microgravity, it has also been found that the killing activity of human primary NK cells and NK-92 cell line were decreased significantly after being cultured in SMG for 48 and 72 h, respectively [43]. These studies all indicate that the microgravity environment has a certain inhibitory effect on the killing activity of NK cells. However, there remains a controversy about the effect of microgravity on the function of NK cells. Buravkova *et al*. treated NK cells with microgravity for 24 h on the ISS and studied the changes of their interaction with target cells. Although changes in the expressions of cytoskeletal and adhesion molecules were observed under microgravity conditions, *in vitro* NK cells maintained their ability to contact, recognize and destroy cancer cells; this suggests that the interaction between the NK cells and target cells was not impaired, thus preserving the critical physiological function of immune cells [114]. Similarly, some studies have also found that the function of NK cells did not change after microgravity simulation via an RWV system for 2–8 days [44]. The reasons for the inconsistent results of these studies may be related to the different conditions and durations of space flight or ground-based SMG, or the use of different cellular models and the different states in which the cells were placed in the different devices under real microgravity and SMG (figure 7). In fact, there is a lack of adequate research to explain these controversies. Overall, although the research results of the effects of microgravity on NK cells are inconsistent, most of the current reports indicate that the killing ability of NK cells is significantly inhibited under microgravity conditions.

Regarding the research on the mechanism by which microgravity affects NK cells, Mylabathula *et al*. used an RCCS to expose human peripheral blood mononuclear cells to SMG for 12 h; they found that the killing activity of NK cells against tumour cells K562 and U266 was reduced by 50%, and the expressions of NK cell perforin and granzyme B were also significantly reduced [115]. However, inconsistent results have also been obtained. For instance, Bigley *et al*. found that microgravity did not affect the *in vitro* degranulation of NK cell perforin and granzyme B in blood samples from crew members on a six-month mission to the ISS [23]. In addition, activating and inhibitory receptors on the surface of NK cells produce activating and inhibitory signals, respectively, and the strengths of these two signals determine the final function of NK cells. Therefore, changes in the expression of the relevant receptors can significantly affect the final function of NK cells. Similarly, there are inconsistent findings for NK cell surface receptor expression. For example, Mylabathula *et al*. used an RCCS to simulate microgravity to treat human peripheral blood mononuclear cells, and found no significant changes in the expression of NK cell surface activating receptors such as NKG2D and NKp30 and inhibitory receptors such as NKG2A and KLRG1 [115]. However, Li *et al*. used a 2D-RWV to simulate a microgravity environment, and found that the expressions of activating receptors NKp30 and NKp44 on the surface of NK cells were not affected, but the expressions of activating receptor NKG2D and inhibitory receptor NKG2A were significantly downregulated [38]. The authors’ former studies showed that the significant downregulation of the activated receptor NKG2D and the significant upregulation of an important inhibitory receptor CD158a may represent an important explanation for the inhibition of cytotoxicity in NK cells by SMG [43]. The *in vitro*-expanded NK cell line used by the authors of the present review and Li *et al*. require exogenous cytokines such as IL-2 to maintain growth and activity, whereas Mylabathula *et al*. used primary undifferentiated NK cells; this may have contributed to the inconsistent findings. Therefore, subsequent studies may also have to consider the effect of cytokines or other exogenous factors. In addition, in the authors' former studies (figure 7) [43], it was further investigated whether DNA methylation participates in NKG2D gene regulation in NK cells within SMG. Although the results obtained were negative, they still provide important information and inspiration for an in-depth understanding of the regulatory mechanisms of NK cell cytotoxicity. It was also found that the downregulation of DAP10 expression was the possible cause of the downregulation of NKG2D expression by SMG and the subsequent suppression of NK cell activity. However, the inhibition of DAP10 expression by SMG was also not achieved by influencing methylation, and thus, the in-depth mechanism still requires further exploration. The decreased activity of NK cells is closely related to the increased risk of viral infection and multiple cancers, so the normal function of NK cells has important practical significance for long-term space missions [116,117]. However, at present, there is a lack of in-depth analysis of the immune function of NK cells, which requires further investigation.

## 3. Recovery measures for damaged immune cells in the microgravity environment

To summarize the studies described previously, it is clear that the microgravity environment in space can negatively affect the immune cell function of the human body during both long-term and short-term space flights, thus posing a serious threat to the health of astronauts. Therefore, the research and development of drugs for the prevention and treatment of damaged immune cells of astronauts and post-flight recovery drugs are particularly important. At present, the relevant reports on the means of damage reversal mainly focus on the activation of some signalling pathways and some natural immune cell activators.

### 3.1. Activation of related signalling pathways

NF-κB plays an important role in controlling gene transcription, cytokine production, etc. [118]. Summarizing the existing studies, it was found that the NF-κB signalling pathway in a variety of immune cells is affected under microgravity conditions [119]. NF-κB is activated in response to external stimuli such as inflammation, oxidative stress, etc., leading to changes in downstream stress response genes [120,121]. For example, Shi *et al*. found that the lack of macrophage production and the impaired function of macrophages in the microgravity environment may be caused by their mediating signalling pathways, such as RAS, ERK, NF-κB and p53. Under the influence of these pathways, the expression of some metabolic genes and proteins is further downregulated [26], which affects the immune function of macrophages. Zhao *et al*. also found that SMG promotes apoptosis by co-regulating Uev1A/TICAM/TRAF/NF-κB-regulated apoptosis and the p53/PCNA- and ATM/ATR-Chk1/2-controlled DNA damage response pathways [78]. All these findings indicate that the NF-κB signalling pathway plays an important role in the proliferation, activation, metabolism and apoptosis of immune cells. Therefore, the development of drugs that act on this signalling pathway will play an important role in the prevention and treatment of immune system damage caused by microgravity. Previous studies have found that exogenous ERK and NF-κB activators significantly counteracted the effect of microgravity on macrophage differentiation [26]. In addition, IL-12 and IL-15, as activating cytokines for NK cells, play a key role in NK cell growth and development [46,47], and they can act through a variety of signalling pathway networks, including NF-κB [122], Li *et al*. also found that IL-15 either alone or in combination with IL-12 could antagonize the inhibition of the killing activity of NK cells caused by SMG. Thus, the combination of IL-12 and IL-15 could be a therapeutic strategy to overcome the effects of microgravity on human NK cells during long-term space missions [38]. Considering that NF-κB is commonly affected in immune cells, future research is necessary for the development of more drugs that act on this signalling pathway, and attention should also be paid to whether the drugs can have negative effects on the organism while enhancing the function of the immune system.

### 3.2. Immune cell activators

Natural compounds such as polysaccharides, polyphenols, flavonoids and saponins have been shown to have immunomodulatory effects. They also have the advantages of multiple targets and high safety, thus occupying an important position in drug development [123]. However, the regulation of immune cells by these compounds in microgravity may not be the same as that in normal gravity. Therefore, it is of great significance to study the effects of natural compounds on immune cells under microgravity conditions, and to screen out compounds that have positive regulatory effects on the recovery of immune impairment under microgravity conditions. Summarizing the available literature, it was found that these natural compounds mitigate the effects of microgravity mainly in terms of affecting immune cell proliferation, apoptosis and function.

#### 3.2.1. Interventions for cell proliferation

It has been found that some synthetic formamides or polysaccharides derived from plants or fungi can regulate the proliferation and activation of immune cells by enhancing the expression of cytokines and surface receptors, and they are strong immunostimulants [124,125]. For example, the synthesis of benzofuran-2-carboxylic acid derivatives (KMEG) can promote lymphocyte proliferation and have a certain protective effect on immune cells exposed to microgravity [48]. Moreover, 78 differentially expressed genes have been found in KMEG-treated normal human peripheral blood mononuclear cells (lymphocytes) after space flight, among which six were found to be significantly upregulated and five were found to be significantly downregulated. These genes play important roles in promoting T lymphocyte proliferation, regulating membrane transport and promoting anti-apoptotic activity [126]. These studies show that KMEG can promote T cell proliferation and reduce T cell apoptosis in the microgravity environment, thereby improving the body's immune function. In addition, polysaccharides from mulberry leaves and *Cordyceps sinensis* mycelium have been found to stimulate the proliferation of immune cells under normal gravity [127]. Some sulfated polysaccharides extracted from seaweed can also promote the proliferation and activation of immunocompetent cells by enhancing the expression of cytokines and surface receptors [128]. However, whether these polysaccharides can also stimulate cell proliferation in the microgravity environment remains to be further studied.

#### 3.2.2. Recovery against apoptosis

Studies have found that microgravity can aggravate cell apoptosis by promoting intracellular ROS generation, thereby inducing the activation of the ROS-sensitive ERK/MKP-1/caspase-3 pathway [49]. N-acetylcysteine (NAC) and quercetin act as antioxidants and can inhibit the continuous phosphorylation of ERK, MKP-1 expression, and the caspase-3 activation of cells, which will reverse the apoptosis of B cells in the microgravity environment and maintain normal proliferation. Therefore, NAC and quercetin may be good candidates for the protection of the health and safety of astronauts and space travellers [49]. In addition, polysaccharides such as *Ganoderma lucidum* polysaccharide (GLP), *Lycium barbarum* polysaccharide (LBP) and *Lentinan* polysaccharide (LNT) can also protect NK cells by inhibiting the apoptosis and necrosis induced by microgravity [50]. Therefore, a future research direction is to screen out drugs from antioxidants and polysaccharides that antagonize the inhibitory effect of microgravity on immune cells. With the further development of the space industry, this research will become increasingly more important.

#### 3.2.3. Recovery against impaired function

In addition to phagocytosis, macrophages can also secrete some cytokines and inflammatory mediators such as NO, TNF-α, IL-1, IL-6, etc. The microgravity environment will lead to macrophage cytokine secretion disorders, such as the decreased secretion of NO, TNF-α, IL-6, etc. [41,103]. Morin (3, 5, 7, 2′, 4‘-pentahydroxyflavone) is a flavonoid component found in many herbs and fruits that has antioxidant properties [129,130] and anti-inflammatory effects [131]. Morin has been found to alleviate inflammation by reducing the secretion of cytokines and inflammatory factors. However, opposite results have been obtained in microgravity, and one study found that morin sulfates/glucuronides can effectively compensate for the impaired macrophage function in microgravity simulated by a three-dimensional culture system using RWV [51]. However, the relevant mechanism of action is not clear, and it is speculated that it may be related to the ability of morin to activate or inhibit protein phosphorylation, thereby regulating the expression of certain genes and ultimately preventing the desensitization of macrophages under microgravity conditions [51]. In addition, polysaccharides such as GLP, LBP and LNT can also restore the function of NK cells damaged under SMG conditions by increasing the killing activity of NK cells and upregulating the expressions of NKG2D and NKp30; this restorative effect may be mediated by the polysaccharide targeting of TLR-4 and CR3 [50]. Follow-up studies could focus on revealing the exact mechanism while developing multiple similar drugs to boost the immunity of astronauts.

## 4. Summary and future directions

Immune cells are very sensitive to the effect of microgravity, which can lead to damage to the body's immune system, thus making astronauts vulnerable to pathogens and significantly increasing the incidence of various diseases that pose a serious threat to the health of astronauts. Therefore, it is of great significance to understand the impact of changes in gravity on immune cells, and to evaluate the mechanism by which immune cells adapt to microgravity.

In summary of the existing studies, the microgravity environment has a significant impact on the whole development process of immune cells, including their proliferation, differentiation, activation, metabolism, structure, etc. and thereby affects the normal function of immune cells. First, in terms of the number of immune cells, microgravity can significantly inhibit the differentiation of monocytes and haematopoietic stem cells into macrophages, resulting in a significant decrease in the number of macrophages. However, the number of neutrophils is increased in the microgravity environment, which may be due to IL-8 inducing the bone marrow to release more neutrophils. Most studies have pointed out that microgravity can reduce the overall number of immune cells by inhibiting the differentiation of DCs and promoting the apoptosis of NK cells and B cells. However, contradictory results still exist, which may be due to the different methods and durations of microgravity generation and the origin of cell species.

In terms of immune cell activation, current research mainly focuses on DCs and T cells. The effect of microgravity on the activation of DCs is time-dependent. Short-term microgravity treatment can promote their development, but when exposed to microgravity for a long period, DCs may be damaged. Moreover, microgravity can inhibit the antigen presentation of DCs to T cells by inhibiting the secretion of IL-12 and other cytokines [32]. Based on this, T cell function may be significantly inhibited in microgravity. Furthermore, microgravity also inhibits the secretion of certain cytokines such as IL-2, leading to the impaired activation of T cells. Additionally, alterations in the translocation and activation of PKC may be another important reason for the decrease of T cell activity. However, how PKC changes and cytoskeleton interact with each other under microgravity, and thereby influenced T cell function remain unclear and must be further studied. In addition, ElGindi *et al*. found that when compared with two-dimensional cell culture, three-dimensional cell culture attenuated the effects of simulated microgravity on the T cell transcriptome and nuclear irregularities, and simulated microgravity appeared to have less effect on activated T cells than resting T cells [132]. This also suggests that we should fully consider the effects of the culture model of T cells (two- or three-dimensional) and the activation state of the cells, so as to give a more authentic reflection on the effects of simulated microgravity on T cells in their native environment.

Relatively little research has been conducted on immune cell metabolism, and previous research has mainly focused on macrophages. Microgravity can reprogramme the metabolism of macrophages, resulting in different degrees of changes in lipid metabolism, nucleotide metabolism and other metabolic pathways, which then lead to a decrease in the ability of macrophages to defend against microbial infection. However, there has been no report on the specific mechanism by which microgravity affects the metabolic pathways of macrophages, which may be an emerging and promising research direction and will provide an important theoretical basis for screening relevant targeted drugs.

In terms of immune cell structure and function, microgravity can downregulate the expression of surface molecules related to the phagocytosis and migration of monocytes, and can change the distribution of cytoskeleton proteins in the cell, thus leading to impaired motor function. It also causes translocation and reduction of the number of various isoforms of PKC in monocytes, which may then affect the cell activation, differentiation, apoptosis and cell cycle processes associated with PKC [80,84]. Additionally, for macrophages, microgravity can lead to the reduction in the expression of adhesion molecules and disturbances to the release of cytokines, which will lead to damage to macrophage migration, the activation of innate immune responses, oxidative bursts and other functions. In addition, short-term microgravity may upregulate the expression of adhesion molecules in neutrophils, while long-term microgravity may decrease the capacity of phagocytosis and oxidative burst. For NK cells, microgravity significantly reduces the expression of killing particles such as perforin and granzyme and their surface activating receptors and upregulates the expression of inhibitory receptors, which thereby affects their killing function. However, the findings are also controversial, and may be related to the different conditions and durations of space flight or ground-based SMG, as well as the interference of certain exogenous factors.

Overall, microgravity may have effects on various aspects of different immune cells. However, the specific effects are closely related to the device and duration of real microgravity or SMG, the cell species, and the interference of external cytokines and other factors. These have led to contradictory results of the effects of microgravity on various aspects of immune cells. Therefore, the influences of these factors should be fully considered, and the interference factors should be eliminated in future ground-based experiments to achieve the same standard as the real microgravity environment to the greatest possible extent. In addition, based on the current research, the potential projects that require further study include the following: (i) significant changes in multiple cytoskeletal networks have been observed under both space flight and ground-based SMG conditions [133], resulting in changes in cell morphology, size, volume and adhesion properties [134–136]. Therefore, cytoskeletal proteins are considered potential gravity sensors, and the study of cytoskeletal change patterns under microgravity conditions is important for understanding the effects of microgravity on immune cells. Microgravity has been found to lead to changes in the number and distribution of cytoskeletal proteins and cytoskeleton-related genes, but further studies on the regulatory mechanism at the signalling pathway level are lacking. In addition, it is not clear how the changes in PKC and the regulation of ICAM-1 interact with the cytoskeleton to affect immune cell function, and, therefore, the specific mechanism by which microgravity inhibits immune cell function should be further analysed from the perspective of the effect on cytoskeletal changes. (ii) The effect of microgravity on a certain immune cell may sometimes act through other immune cells. Therefore, when studying the effects of microgravity on the immune system, not only should the effects on a single cell be considered, but the synergistic effect between different immune cells should also be taken into account to truly reflect the mechanism by which microgravity affects human health. This may also be an important direction for future related research.

Finally, the development of related drugs that can reverse the previously mentioned damage to the immune system in microgravity is critical to defending against the health threats posed by microgravity to astronauts. On the one hand, current reports about the means of reversal focus on drugs that target the activation of signalling pathways, such as NF-κB. NF-κB agonists, exogenous ERK agonists and IL-15 alone or in combination with IL-12 can partially reverse the negative effects of microgravity on immune cells by targeting this signalling pathway. Therefore, it is of great significance for the development of related reversal drugs to reveal which signal pathways are regulated by microgravity and then affect immune cells. On the other hand, some natural immune cell activators such as polysaccharides, polyphenols, flavonoids, saponins and other natural compounds can, respectively, target the proliferation, apoptosis and function of immune cells to alleviate the effects of microgravity. However, the specific mechanism of the effects of these natural compounds remains unclear. Therefore, future studies can consider this as the main research direction to screen new targets, and then to develop a variety of related drugs to improve the immune function of astronauts and ensure their health.

In summary, more studies are required to reveal the effect mechanism of microgravity on immune cells and develop more effective and safe drugs for ensuring the health of astronauts.

## Data Availability

This article has no additional data.

## References

[1] Sonnenfeld G, Shearer WT. 2002 Immune function during space flight. Nutrition **18**, 899-903. (10.1016/S0899-9007(02)00903-6) 12361785

[2] Garrett-Bakelman FE et al. 2019 The NASA twins study: a multidimensional analysis of a year-long human spaceflight. Science **364**, eaau8650. (10.1126/science.aau8650) 30975860 PMC7580864

[3] Cogoli A, Tschop A. 1985 Lymphocyte reactivity during spaceflight. Immunol. Today **6**, 1-4. (10.1126/science.6729481) 11539785

[4] Hodgkins JE, Reeves WP. 1964 The modified kaluza synthesis. III. The synthesis of some aromatic isothiocyanates. J. Org. Chem. **29**, 3098-3099. (10.1021/jo01033a524)

[5] Bradbury P, Wu H, Choi JU, Rowan AE, Zhang H, Poole K, Lauko J, Chou J. 2020 Modeling the impact of microgravity at the cellular level: implications for human disease. Front. Cell Dev. Biol. **8**, 96. (10.3389/fcell.2020.00096) 32154251 PMC7047162

[6] Crucian BE et al*.* 2018 Immune system dysregulation during spaceflight: potential countermeasures for deep space exploration missions. Front. Immunol. **9**, 1437. (10.3389/fimmu.2018.01437) 30018614 PMC6038331

[7] Guilak F, Tedrow JR, Burgkart R. 2000 Viscoelastic properties of the cell nucleus. Biochem. Biophys. Res. Commun. **269**, 781-786. (10.1006/bbrc.2000.2360) 10720492

[8] Delves PJ, Roitt IM. 2000 The immune system. Adv. Immunol. **343**, 37-50. 10.1056/NEJM200007063430107 10882768

[9] Guéguinou N, Huin-Schohn C, Bascove M, Bueb J-L, Tschirhart E, Legrand-Frossi C, Frippiat J-P. 2009 Could spaceflight-associated immune system weakening preclude the expansion of human presence beyond Earth's orbit? J. Leukoc. Biol. **86**, 1027-1038. (10.1189/jlb.0309167) 19690292

[10] Ullrich O, Huber K, Lang K. 2008 Signal transduction in cells of the immune system in microgravity. Cell Commun. Signal. **6**, 6. (10.1186/1478-811X-6-9) 18957108 PMC2583999

[11] Zhu L et al. 2021 Attenuation of antiviral immune response caused by perturbation of TRIM25-mediated RIG-I activation under simulated microgravity. Cell Rep. **34**, 108600. (10.1016/j.celrep.2020.108600) 33406425

[12] Taylor PW, Sommer AP. 2005 Towards rational treatment of bacterial infections during extended space travel. Int. J. Antimicrob. Agents **26**, 183-187. (10.1016/j.ijantimicag.2005.06.002) 16118047 PMC2025679

[13] Durnova GN, Kaplansky AS, Portugalov VV. 1976 Effect of a 22-day space flight on the lymphoid organs of rats. Aviat. Space Environ. Med. **47**, 588-591. 938393

[14] Baqai FP, Gridley DS, Slater JM, Luo-Owen X, Stodieck LS, Ferguson V, Chapes SK, Pecaut MJ. 2009 Effects of spaceflight on innate immune function and antioxidant gene expression. J. Appl. Physiol. **106**, 1935-1942. (10.1152/japplphysiol.91361.2008) 19342437 PMC2692779

[15] Gridley DS et al. 2003 Selected contribution: effects of spaceflight on immunity in the C57BL/6 mouse. II. activation, cytokines, erythrocytes, and platelets. J. Appl. Physiol. **94**, 2095-2103. (10.1152/japplphysiol.01053.2002) 12506046

[16] Crucian B, Babiak-Vazquez A, Johnston S, Pierson DL, Ott CM, Sams C. 2016 Incidence of clinical symptoms during long-duration orbital spaceflight. Int. J. Gen. Med. **9**, 383-391. (10.2147/IJGM.S114188) 27843335 PMC5098747

[17] Crucian B, Sams C. 2009 Immune system dysregulation during spaceflight: clinical risk for exploration-class missions. J. Leukoc. Biol. **86**, 1017-1018. (10.1189/jlb.0709500) 19875627

[18] Zayzafoon M, Meyers VE, McDonald JM. 2005 Microgravity: the immune response and bone. Immunol. Rev. **208**, 267-280. (10.1111/j.0105-2896.2005.00330.x) 16313354

[19] Sonnenfeld G. 1999 Space flight, microgravity, stress, and immune responses. Adv. Space Res. Off. J. Comm. Space Res. COSPAR **23**, 1945-1953. (10.1016/S0273-1177(99)00455-X) 11710376

[20] Crucian B, Stowe R, Mehta S, Uchakin P, Quiriarte H, Pierson D, Sams C. 2013 Immune system dysregulation occurs during short duration spaceflight on board the space shuttle. J. Clin. Immunol. **33**, 456-465. (10.1007/s10875-012-9824-7) 23100144

[21] Morabito C, Lanuti P, Caprara GA, Marchisio M, Bizzarri M, Guarnieri S, Mariggiò MA. 2019 Physiological responses of Jurkat lymphocytes to simulated microgravity conditions. Int. J. Mol. Sci. **20**, 1892. (10.3390/ijms20081892) 30999563 PMC6515345

[22] Pellis NR, Goodwin TJ, Risin D, McIntyre BW, Pizzini RP, Cooper D, Baker TL, Spaulding GF. 1997 Changes in gravity inhibit lymphocyte locomotion through type I collagen. Vitro Cell. Dev. Biol. Anim. **33**, 398-405. (10.1007/s11626-997-0012-7) 9196900

[23] Bigley AB et al. 2019 NK cell function is impaired during long-duration spaceflight. J. Appl. Physiol. **126**, 842-853. (10.1152/japplphysiol.00761.2018) 30382809

[24] Taylor GR, Konstantinova I, Sonnenfeld G, Jennings R. 1997 Chapter 1 changes in the immune system during and after spaceflight. In Advances in space biology and medicine, pp. 1-32. 10.1016/s1569-2574(08)60076-3 9048132

[25] Kaur I, Simons ER, Castro VA, Mark Ott C, Pierson DL. 2004 Changes in neutrophil functions in astronauts. Brain. Behav. Immun. **18**, 443-450. (10.1016/j.bbi.2003.10.005) 15265537

[26] Shi L, Tian H, Wang P, Li L, Zhang Z, Zhang J, Zhao Y. 2021 Spaceflight and simulated microgravity suppresses macrophage development via altered RAS/ERK/NF*κ*B and metabolic pathways. Cell. Mol. Immunol. **18**, 1489-1502. (10.1038/s41423-019-0346-6) 31900461 PMC8167113

[27] Adrian A, Schoppmann K, Sromicki J, Brungs S, von der Wiesche M, Hock B, Kolanus W, Hemmersbach R, Ullrich O. 2013 The oxidative burst reaction in mammalian cells depends on gravity. Cell Commun. Signal. **11**, 98. (10.1186/1478-811X-11-98) 24359439 PMC3880029

[28] Radulovic M, Godovac-Zimmermann J. 2011 Proteomic approaches to understanding the role of the cytoskeleton in host-defense mechanisms. Expert Rev. Proteomics **8**, 117-126. (10.1586/epr.10.91) 21329431 PMC4261605

[29] Low EK, Brudvik E, Kuhlman B, Wilson PF, Almeida-Porada G, Porada CD. 2018 Microgravity impairs DNA damage repair in human hematopoietic stem/progenitor cells and inhibits their differentiation into dendritic cells. Stem Cells Dev. **27**, 1257-1267. (10.1089/scd.2018.0052) 29901426

[30] Monici M, Basile V, Bellik L, Fusi F, Marziliano N, Parenti A, Romano G, Conti A. 2007 Does the exposure to microgravity affect dendritic cell maturation from monocytes? Microgravity Sci. Technol. **19**, 187-190. (10.1007/BF02919479)

[31] Chen H, Luo H, Liu J, Wang P, Dong D, Shang P, Zhao Y. 2015 The distinctive sensitivity to microgravity of immune cell subpopulations. Microgravity Sci. Technol. **27**, 427-436. (10.1007/s12217-015-9441-1)

[32] Savary CA, Grazziutti M, Tomasovic SR, Mcintyre B, Woodside DG, Pellis N, Pierson DL, Rex AH. 2001 Characteristics of human dendritic cells generated in a microgravity analog culture system. Vitro Cell Dev. Biol. Anim. **37**, 216-222. (10.1007/BF02577532) 11409686

[33] Risso A, Tell G, Vascotto C, Costessi A, Arena S, Scaloni A, Cosulich ME. 2005 Activation of human T lymphocytes under conditions similar to those that occur during exposure to microgravity: a proteomics study. Proteomics **5**, 1827-1837. (10.1002/pmic.200401082) 15825147

[34] Wang C, Li N, Zhang C, Sun S, Gao Y, Long M. 2015 Effects of simulated microgravity on functions of neutrophil-like HL-60 Cells. Microgravity Sci. Technol. **27**, 515-527. (10.1007/s12217-015-9473-6)

[35] Tascher G et al. 2019 Analysis of femurs from mice embarked on board bion-M1 biosatellite reveals a decrease in immune cell development, including B cells, after 1 wk of recovery on Earth. FASEB J. **33**, 3772-3783. (10.1096/fj.201801463R) 30521760

[36] Spielmann G, Agha N, Kunz H, Simpson RJ, Crucian B, Mehta S, Laughlin M, Campbell J. 2019 B cell homeostasis is maintained during long-duration spaceflight. J. Appl. Physiol. Bethesda Md 1985 **126**, 469-476. (10.1152/japplphysiol.00789.2018) PMC6397409 30496712

[37] Crucian B, Stowe R, Quiriarte H, Pierson D, Sams C. 2011 Monocyte phenotype and cytokine production profiles are dysregulated by short-duration spaceflight. Aviat. Space Environ. Med. **82**, 857-862. (10.3357/asem.3047.2011) 21888268

[38] Li Q, Mei Q, Huyan T, Xie L, Che S, Yang H, Zhang M, Huang Q. 2013 Effects of simulated microgravity on primary human NK cells. Astrobiology **13**, 703-714. (10.1089/ast.2013.0981) 23919749 PMC3746215

[39] Gridley DS, Slater JM, Luo-Owen X, Rizvi A, Chapes SK, Stodieck LS, Ferguson VL, Pecaut MJ. 2009 Spaceflight effects on T lymphocyte distribution, function and gene expression. J. Appl. Physiol. **106**, 194-202. (10.1152/japplphysiol.91126.2008) 18988762 PMC2636934

[40] Felix K, Wise K, Manna S, Yamauchi K, Wilson BL, Thomas RL, Kulkarni A, Pellis NR, Ramesh GT. 2004 Altered cytokine expression in tissues of mice subjected to simulated microgravity. Mol. Cell. Biochem. **266**, 79-85. (10.1023/B:MCBI.0000049136.55611.dd) 15646029

[41] Kaur I, Simons ER, Castro VA, Ott CM, Pierson DL. 2005 Changes in monocyte functions of astronauts. Brain. Behav. Immun. **19**, 547-554. (10.1016/j.bbi.2004.12.006) 15908177

[42] Kaur I, Simons ER, Kapadia AS, Ott CM, Pierson DL. 2008 Effect of spaceflight on ability of monocytes to respond to endotoxins of gram-negative bacteria. Clin. Vaccine Immunol. **15**, 1523-1528. (10.1128/CVI.00065-08) 18768671 PMC2565938

[43] Shao D, Ye L, Zhu B, Li Q, Yang H, Shi J, Huang Q, Zhao W. 2021 Mechanisms of the effect of simulated microgravity on the cytotoxicity of NK cells following the DNA methylation of NKG2D and the expression of DAP10. Microgravity Sci. Technol. **33**, 6. (10.1007/s12217-020-09863-3)

[44] Licato LL, Grimm EA. 1999 Multiple interleukin-2 signaling pathways differentially regulated by microgravity. Immunopharmacology **44**, 273-279. (10.1016/S0162-3109(99)00123-X) 10598884

[45] Stervbo U et al. 2018 Gravitational stress during parabolic flights reduces the number of circulating innate and adaptive leukocyte subsets in human blood. PloS ONE **13**, e0206272. (10.1371/journal.pone.0206272) 30427865 PMC6235284

[46] Liu E et al. 2018 Cord blood NK cells engineered to express IL-15 and a CD19-targeted CAR show long-term persistence and potent antitumor activity. Leukemia **32**, 520-531. (10.1038/leu.2017.226) 28725044 PMC6063081

[47] Abel AM, Yang C, Thakar MS, Malarkannan S. 2018 Natural killer cells: development, maturation, and clinical utilization. Front. Immunol. **9**, 1869. (10.3389/fimmu.2018.01869) 30150991 PMC6099181

[48] Sundaresan A, Marriott K, Mao J, Bhuiyan S, Denkins P. 2015 The effects of benzofuran-2-carboxylic acid derivatives as countermeasures in immune modulation and cancer cell inhibition. Microgravity Sci. Technol. **27**, 129-140. (10.1007/s12217-014-9408-7)

[49] Dang B et al. 2014 Simulated microgravity increases heavy ion radiation-induced apoptosis in human B lymphoblasts. Life Sci. **97**, 123-128. (10.1016/j.lfs.2013.12.008) 24361401

[50] Huyan T, Li Q, Yang H, Jin M-L, Zhang M-J, Ye L-J, Li J, Huang Q-S, Yin D-C. 2014 Protective effect of polysaccharides on simulated microgravity-induced functional inhibition of human NK cells. Carbohydr. Polym. **101**, 819-827. (10.1016/j.carbpol.2013.10.021) 24299844

[51] Hsieh C-L, Chao P-DL, Fang S-H. 2005 Morin sulphates/glucuronides enhance macrophage function in microgravity culture system. Eur. J. Clin. Invest. **35**, 591-596. (10.1111/j.1365-2362.2005.01551.x) 16128866

[52] Singh N, Baby D, Rajguru JP, Patil PB, Thakkannavar SS, Pujari VB. 2019 Inflammation and cancer. Ann. Afr. Med. **18**, 121-126. (10.4103/aam.aam_56_18) 31417011 PMC6704802

[53] Racine RN, Cormier SM. 1992 Effect of spaceflight on rat hepatocytes: a morphometric study. J. Appl. Physiol. Bethesda Md 1985 **73**, 136S-141S. 10.1152/jappl.1992.73.2.S136 1526940

[54] Hou Y, Zhu L, Tian H, Sun H-X, Wang R, Zhang L, Zhao Y. 2018 IL-23-induced macrophage polarization and its pathological roles in mice with imiquimod-induced psoriasis. Protein Cell **9**, 1027-1038. (10.1007/s13238-018-0505-z) 29508278 PMC6251802

[55] Zhu L et al. 2014 TSC1 controls macrophage polarization to prevent inflammatory disease. Nat. Commun. **5**, 4696. (10.1038/ncomms5696) 25175012

[56] Sawyer DW, Donowitz GR, Mandell GL. 1989 Polymorphonuclear neutrophils: an effective antimicrobial force. Clin. Infect. Dis. **11**, S1532-S1544. (10.1093/clinids/11.Supplement_7.S1532) 2557663

[57] Stowe RP, Sams CF, Mehta SK, Kaur I, Jones ML, Feeback DL, Pierson DL. 1999 Leukocyte subsets and neutrophil function after short-term spaceflight. J. Leukoc. Biol. **65**, 179-186. (10.1002/jlb.65.2.179) 10088600

[58] Kim M, Jang G, Kim K-S, Shin J. 2022 Detrimental effects of simulated microgravity on mast cell homeostasis and function. Front. Immunol. **13**, 1055531. (10.3389/fimmu.2022.1055531) 36591304 PMC9800517

[59] Kaufmann I, Schachtner T, Feuerecker M, Schelling G, Thiel M, Choukèr A. 2009 Parabolic flight primes cytotoxic capabilities of polymorphonuclear leucocytes in humans. Eur. J. Clin. Invest. **39**, 723-728. (10.1111/j.1365-2362.2009.02136.x) 19473213

[60] Paul AM, Mhatre SD, Cekanaviciute E, Schreurs A-S, Tahimic CGT, Globus RK, Anand S, Crucian BE, Bhattacharya S. 2020 Neutrophil-to-lymphocyte ratio: a biomarker to monitor the immune status of astronauts. Front. Immunol. **11**, 564950. (10.3389/fimmu.2020.564950) 33224136 PMC7667275

[61] Loos T, Opdenakker G, Van Damme J, Proost P. 2009 Citrullination of CXCL8 increases this chemokine's ability to mobilize neutrophils into the blood circulation. Haematologica **94**, 1346-1353. (10.3324/haematol.2009.006973) 19608678 PMC2754949

[62] Van Eeden SE, Terashima T. 2000 Interleukin 8 (IL-8) and the release of leukocytes from the bone marrow. Leuk. Lymphoma **37**, 259-271. (10.3109/10428190009089427) 10752978

[63] Hu W, Wang G, Huang D, Sui M, Xu Y. 2019 Cancer immunotherapy based on natural killer cells: current progress and new opportunities. Front. Immunol. **10**, 1205. (10.3389/fimmu.2019.01205) 31214177 PMC6554437

[64] Herberman RB, Nunn ME, Holden HT, Lavrin DH. 1975 Natural cytotoxic reactivity of mouse lymphoid cells against syngeneic and allogeneic tumors. II. Characterization of effector cells. Int. J. Cancer **16**, 230-239. (10.1002/ijc.2910160205) 1080480

[65] Liu W, Zhu X, Zhao L, Yang X, Cao F, Huang Y, Mu P. 2015 Effects of simulated weightlessness on biological activity of human NK cells induced by IL-2. Chin. J. Cell. Mol. Immunol. **31**, 1297-1305. 26429526

[66] Helmink B et al. 2020 B cells and tertiary lymphoid structures promote immunotherapy response. Nature **577**, 549-555. (10.1038/s41586-019-1922-8) 31942075 PMC8762581

[67] Banchereau J, Steinman RM. 1998 Dendritic cells and the control of immunity. Nature **392**, 245-252. (10.1038/32588) 9521319

[68] Tackett N, Bradley JH, Moore EK, Baker SH, Minter SL, DiGiacinto B, Arnold JP, Gregg RK. 2019 Prolonged exposure to simulated microgravity diminishes dendritic cell immunogenicity. Sci. Rep. **9**, 13825. (10.1038/s41598-019-50311-z) 31554863 PMC6761163

[69] Spatz JM, Fulford MH, Tsai A, Gaudilliere D, Hedou J, Ganio E, Angst M, Aghaeepour N, Gaudilliere B. 2021 Human immune system adaptations to simulated microgravity revealed by single-cell mass cytometry. Sci. Rep. **11**, 11872. (10.1038/s41598-021-90458-2) 34099760 PMC8184772

[70] Luo H, Wang C, Feng M, Zhao Y. 2014 Microgravity inhibits resting T cell immunity in an exposure time-dependent manner. Int. J. Med. Sci. **11**, 87-96. (10.7150/ijms.7651) 24396290 PMC3880995

[71] Paulsen K et al. 2010 Microgravity-induced alterations in signal transduction in cells of the immune system. Acta Astronaut. **67**, 1116-1125. (10.1016/j.actaastro.2010.06.053)

[72] Martinez EM, Yoshida MC, Candelario TLT, Hughes-Fulford M. 2015 Spaceflight and simulated microgravity cause a significant reduction of key gene expression in early T-cell activation. Am. J. Physiol. Regul. Integr. Comp. Physiol. **308**, R480-R488. (10.1152/ajpregu.00449.2014) 25568077 PMC4360066

[73] Liao W, Lin J-X, Leonard WJ. 2013 Interleukin-2 at the crossroads of effector responses, tolerance, and immunotherapy. Immunity **38**, 13-25. (10.1016/j.immuni.2013.01.004) 23352221 PMC3610532

[74] Walther I, Pippia P, Meloni MA, Turrini F, Mannu F, Cogoli A. 1998 Simulated microgravity inhibits the genetic expression of interleukin-2 and its receptor in mitogen-activated T lymphocytes. FEBS Lett. **436**, 115-118. (10.1016/S0014-5793(98)01107-7) 9771904

[75] Boonyaratanakornkit JB, Cogoli A, Li C-F, Schopper T, Pippia P, Galleri G, Meloni MA, Hughes-Fulford M. 2005 Key gravity-sensitive signaling pathways drive T-cell activation. FASEB J. **19**, 2020-2022. (10.1096/fj.05-3778fje) 16210397

[76] Ward NE, Pellis NR, Risin SA, Risin D. 2006 Gene expression alterations in activated human T-cells induced by modeled microgravity. J. Cell. Biochem. **99**, 1187-1202. (10.1002/jcb.20988) 16795038

[77] Chang TT, Walther I, Li C, Boonyaratanakornkit J, Galleri G, Meloni MA, Pippia P, Cogoli A, Hughes-Fulford M. 2012 The Rel/NF-*κ*B pathway and transcription of immediate early genes in T cell activation are inhibited by microgravity. J. Leukoc. Biol. **92**, 1133-1145. (10.1189/jlb.0312157) 22750545 PMC3501893

[78] Zhao T, Tang X, Umeshappa CS, Ma H, Gao H, Deng Y, Freywald A, Xiang J. 2016 Simulated microgravity promotes cell apoptosis through suppressing Uev1A/TICAM/TRAF/NF-κB-regulated anti-apoptosis and p53/PCNA- and ATM/ATR-Chk1/2-controlled DNA-damage response pathways. J. Cell. Biochem. **117**, 2138-2148. (10.1002/jcb.25520) 26887372

[79] Cogoli A. 1997 Signal transduction in T lymphocytes in microgravity. Gravitational Space Biol. Bull. **10**, 5-16. 11540120

[80] Hatton JP, Gaubert F, Cazenave J-P, Schmitt D. 2002 Microgravity modifies protein kinase C isoform translocation in the human monocytic cell line U937 and human peripheral blood T-cells. J. Cell. Biochem. **87**, 39-50. (10.1002/jcb.10273) 12210720

[81] Speidel JT, Affandi T, Jones DNM, Ferrara SE, Reyland ME. 2020 Functional proteomic analysis reveals roles for PKC*δ* in regulation of cell survival and cell death: implications for cancer pathogenesis and therapy. Adv. Biol. Regul. **78**, 100757. (10.1016/j.jbior.2020.100757) 33045516 PMC8294469

[82] Saito T, Yokosuka T, Hashimoto-Tane A. 2010 Dynamic regulation of T cell activation and co-stimulation through TCR-microclusters. FEBS Lett. **584**, 4865-4871. (10.1016/j.febslet.2010.11.036) 21110974

[83] Isakov N, Altman A. 2002 Protein kinase C(theta) in T cell activation. Annu. Rev. Immunol. **20**, 761-794. (10.1146/annurev.immunol.20.100301.064807) 11861617

[84] Hatton JP, Gaubert F, Lewis ML, Darsel Y, Ohlmann P, Cazenave J, Schmitt D. 1999 The kinetics of translocation and cellular quantity of protein kinase C in human leukocytes are modified during spaceflight. FASEB J. **13**, S23-S33. (10.1096/fasebj.13.9001.s23) 10352142

[85] Schmitt DA, Hatton JP, Emond C, Chaput D, Paris H, Levade T, Cazenave J, Schaffar L. 1996 The distribution of protein kinase C in human leukocytes is altered in microgravity. FASEB J. **10**, 1627-1634. (10.1096/fasebj.10.14.9002555) 9002555

[86] Larsson C. 2006 Protein kinase C and the regulation of the actin cytoskeleton. Cell. Signal. **18**, 276-284. (10.1016/j.cellsig.2005.07.010) 16109477

[87] Sciola L, Cogoli-Greuter M, Cogoli A, Spano A, Pippia P. 1999 Influence of microgravity on mitogen binding and cytoskeleton in Jurkat cells. Adv. Space Res. **24**, 801-805. (10.1016/S0273-1177(99)00078-2) 11542625

[88] Lewis ML, Cubano LA, Zhao B, Dinh H-K, Pabalan JG, Piepmeier EH, Bowman PD. 2001 cDNA microarray reveals altered cytoskeletal gene expression in space-flown leukemic T lymphocytes (Jurkat). FASEB J. **15**, 1783-1785. (10.1096/fj.00-0820fje) 11481229

[89] Lewis ML, Reynolds JL, Cubano LA, Hatton JP, Lawless BD, Piepmeier EH. 1998 Spaceflight alters microtubules and increases apoptosis in human lymphocytes (Jurkat). FASEB J. **12**, 1007-1018. (10.1096/fasebj.12.11.1007) 9707173

[90] Bradley JH, Barwick S, Horn GQ, Ullrich E, Best B, Arnold JP, Gregg RK. 2019 Simulated microgravity-mediated reversion of murine lymphoma immune evasion. Sci. Rep. **9**, 14623. (10.1038/s41598-019-51106-y) 31602007 PMC6787044

[91] Sonnenfeld G, Mandel AD, Konstantinova IV, Berry WD, Taylor GR, Lesnyak AT, Fuchs BB, Rakhmilevich AL. 1992 Spaceflight alters immune cell function and distribution. J. Appl. Physiol. **73**, S191-S195. (10.1152/jappl.1992.73.2.S191) 1526951

[92] Tauber S et al. 2017 Cytoskeletal stability and metabolic alterations in primary human macrophages in long-term microgravity. PloS ONE **12**, e0175599. (10.1371/journal.pone.0175599) 28419128 PMC5395169

[93] Mráček T, Drahota Z, Houštěk J. 2013 The function and the role of the mitochondrial glycerol-3-phosphate dehydrogenase in mammalian tissues. Biochim. Biophys. Acta **1827**, 401-410. (10.1016/j.bbabio.2012.11.014) 23220394

[94] Carpenter K. 2015 Branched chain amino acids and maple syrup urine disease. In Branched chain amino acids in clinical nutrition (eds R Rajendram, VR Preedy, VB Patel), pp. 145-156. New York, NY: Springer.

[95] Suzuki T, Mower HF, Friesen MD, Gilibert I, Sawa T, Ohshima H. 2004 Nitration and nitrosation of N-acetyl-L-tryptophan and tryptophan residues in proteins by various reactive nitrogen species. Free Radic. Biol. Med. **37**, 671-681. (10.1016/j.freeradbiomed.2004.05.030) 15288124

[96] Wong KL, Tai JJ-Y, Wong W-C, Han H, Sem X, Yeap W-H, Kourilsky P, Wong S-C. 2011 Gene expression profiling reveals the defining features of the classical, intermediate, and nonclassical human monocyte subsets. Blood **118**, e16-e31. (10.1182/blood-2010-12-326355) 21653326

[97] Meloni MA, Galleri G, Pani G, Saba A, Pippia P, Cogoli-Greuter M. 2011 Space flight affects motility and cytoskeletal structures in human monocyte cell line J-111. Cytoskeleton **68**, 125-137. (10.1002/cm.20499) 21246756

[98] Martiny-Baron G, Fabbro D. 2007 Classical PKC isoforms in cancer. Pharmacol. Res. **55**, 477-486. (10.1016/j.phrs.2007.04.001) 17548205

[99] Paulsen K et al. 2014 Severe disruption of the cytoskeleton and immunologically relevant surface molecules in a human macrophageal cell line in microgravity—results of an in vitro experiment on board of the Shenzhou-8 space mission. Acta Astronaut. **94**, 277-292. (10.1016/j.actaastro.2013.06.007)

[100] Paulsen K et al. 2015 Regulation of ICAM-1 in cells of the monocyte/macrophage system in microgravity. BioMed Res. Int. **2015**, 538786. (10.1155/2015/538786) 25654110 PMC4309248

[101] Sun Y, Kuang Y, Zuo Z. 2021 The emerging role of macrophages in immune system dysfunction under real and simulated microgravity conditions. Int. J. Mol. Sci. **22**, 2333. (10.3390/ijms22052333) 33652750 PMC7956436

[102] Wang S, Zhang N, Di J, Zhao W, Shi G, Xie R, Hu B, Yang H. 2021 Analysis of the effects of magnetic levitation to simulate microgravity environment on the Arp2/3 complex pathway in macrophage. J. Biol. Phys. **47**, 323-335. (10.1007/s10867-021-09581-w) 34533653 PMC8452804

[103] Ludtka C, Silberman J, Moore E, Allen JB. 2021 Macrophages in microgravity: the impact of space on immune cells. NPJ Microgravity **7**, 13. (10.1038/s41526-021-00141-z) 33790288 PMC8012370

[104] Wang C, Luo H, Zhu L, Yang F, Chu Z, Tian H, Feng M, Zhao Y, Shang P. 2013 Microgravity inhibition of lipopolysaccharide-induced tumor necrosis factor-α expression in macrophage cells. Inflamm. Res. **63**, 91-98. (10.1007/s00011-013-0676-2) 24196691

[105] Wang C, Chen H, Luo H, Zhu L, Zhao Y, Tian H, Wang R, Shang P, Zhao Y. 2015 Microgravity activates p38 MAPK-C/EBP*β* pathway to regulate the expression of arginase and inflammatory cytokines in macrophages. Inflamm. Res. **64**, 303-311. (10.1007/s00011-015-0811-3) 25804385

[106] Thiel CS et al. 2017 Rapid adaptation to microgravity in mammalian macrophage cells. Sci. Rep. **7**, 43. (10.1038/s41598-017-00119-6) 28242876 PMC5427920

[107] Neefjes J, Jongsma MLM, Paul P, Bakke O. 2011 Towards a systems understanding of MHC class I and MHC class II antigen presentation. Nat. Rev. Immunol. **11**, 823-836. (10.1038/nri3084) 22076556

[108] Yang J, Xing W, Wang C. 2019 Effects of microgravity on the monocyte/macrophage physiology and the potential implication in immune responses to vaccines. Vaccine Res. **6**, 47-52. (10.29252/vacres.6.2.47)

[109] Frippiat J-P et al. 2016 Towards human exploration of space: the THESEUS review series on immunology research priorities. NPJ Microgravity **2**, 16040. (10.1038/npjmgrav.2016.40) 28725745 PMC5515533

[110] Boxer LA, Allen JM, Baehner RL. 1980 Diminished polymorphonuclear leukocyte adherence: function dependent on release of cyclic AMP by endothelial cells after stimulation of beta-receptors by epinephrine. J. Clin. Invest. **66**, 268-274. (10.1172/JCI109853) 6249848 PMC371707

[111] Konstantinova IV. 1991 Immune resistance of man in space flights. Acta Astronaut. **23**, 123-127. (10.1016/0094-5765(91)90108-h) 11537113

[112] Tanaka M, Tonouchi M, Hosono T, Nagashima K, Yanase-Fujiwara M, Kanosue K. 2001 Hypothalamic region facilitating shivering in rats. Jpn. J. Physiol. **51**, 625-629. (10.2170/jjphysiol.51.625) 11734085

[113] Mehta SK, Kaur I, Grimm EA, Smid C, Feeback DL, Pierson DL. 2001 Decreased non-MHC-restricted (CD56^+^) killer cell cytotoxicity after spaceflight. J. Appl. Physiol. **91**, 1814-1818. (10.1152/jappl.2001.91.4.1814) 11568167

[114] Buravkova L, Romanov Y, Rykova M, Grigorieva O, Merzlikina N. 2005 Cell-to-cell interactions in changed gravity: ground-based and flight experiments. Acta Astronaut. **57**, 67-74. (10.1016/j.actaastro.2005.03.012) 16010753

[115] Mylabathula PL et al. 2020 Simulated microgravity disarms human NK-cells and inhibits anti-tumor cytotoxicity in vitro. Acta Astronaut. **174**, 32-40. (10.1016/j.actaastro.2020.03.023)

[116] Vineretsky KA, Karagas MR, Christensen BC, Kuriger-Laber JK, Perry AE, Storm CA, Nelson HH. 2016 Skin cancer risk is modified by KIR/HLA interactions that influence the activation of natural killer immune cells. Cancer Res. **76**, 370-376. (10.1158/0008-5472.CAN-15-0547) 26744525 PMC4715977

[117] Martner A, Rydström A, Riise RE, Aurelius J, Brune M, Foà R, Hellstrand K, Thorén FB. 2015 NK cell expression of natural cytotoxicity receptors may determine relapse risk in older AML patients undergoing immunotherapy for remission maintenance. Oncotarget **6**, 42 569-42 574. (10.18632/oncotarget.5559) PMC4767453 26544512

[118] Oeckinghaus A, Ghosh S. 2009 The NF-κB family of transcription factors and its regulation. Cold Spring Harb. Perspect. Biol. **1**, a000034. (10.1101/cshperspect.a000034) 20066092 PMC2773619

[119] Zhang Y, Moreno-Villanueva M, Krieger S, Ramesh GT, Neelam S, Wu H. 2017 Transcriptomics, NF-κB pathway, and their potential spaceflight-related health consequences. Int. J. Mol. Sci. **18**, E1166. (10.3390/ijms18061166) PMC5485990 28561779

[120] Hayden MS, Ghosh S. 2012 NF-κB, the first quarter-century: remarkable progress and outstanding questions. Genes Dev. **26**, 203-234. (10.1101/gad.183434.111) 22302935 PMC3278889

[121] Hellweg CE. 2015 The nuclear factor *κ*B pathway: a link to the immune system in the radiation response. Cancer Lett. **368**, 275-289. (10.1016/j.canlet.2015.02.019) 25688671

[122] Chenoweth MJ et al. 2012 IL-15 can signal via IL-15R*α*, JNK, and NF-κB to drive RANTES production by myeloid cells. J. Immunol. **188**, 4149-4157. (10.4049/jimmunol.1101883) 22447977

[123] Li Y, Wang X, Ma X, Liu C, Wu J, Sun C. 2021 Natural polysaccharides and their derivates: a promising natural adjuvant for tumor immunotherapy. Front. Pharmacol. **12**, 621813. (10.3389/fphar.2021.621813) 33935714 PMC8080043

[124] Kim G-Y, Oh Y-H, Park Y-M. 2003 Acidic polysaccharide isolated from *Phellinus linteus* induces nitric oxide-mediated tumoricidal activity of macrophages through protein tyrosine kinase and protein kinase C. Biochem. Biophys. Res. Commun. **309**, 399-407. (10.1016/j.bbrc.2003.08.018) 12951063

[125] Leung MYK, Liu C, Koon JCM, Fung KP. 2006 Polysaccharide biological response modifiers. Immunol. Lett. **105**, 101-114. (10.1016/j.imlet.2006.01.009) 16554097

[126] Okoro E, Mann V, Ellis I, Mansoor E, Olamigoke L, Marriott KS, Denkins P, Williams W, Sundaresan A. 2017 Immune modulation in normal human peripheral blood mononuclear cells (PBMCs) (lymphocytes) in response to benzofuran-2-carboxylic acid derivative KMEG during spaceflight. Microgravity Sci. Technol. **29**, 331-336. (10.1007/s12217-017-9551-z)

[127] Wu Y, Sun H, Qin F, Pan Y, Sun C. 2006 Effect of various extracts and a polysaccharide from the edible mycelia of *Cordyceps sinensis* on cellular and humoral immune response against ovalbumin in mice. Phytother. Res. PTR **20**, 646-652. (10.1002/ptr.1921) 16691546

[128] Surayot U, You S. 2017 Structural effects of sulfated polysaccharides from *Codium fragile* on NK cell activation and cytotoxicity. Int. J. Biol. Macromol. **98**, 117-124. (10.1016/j.ijbiomac.2017.01.108) 28130139

[129] Hanasaki Y, Ogawa S, Fukui S. 1994 The correlation between active oxygens scavenging and antioxidative effects of flavonoids. Free Radic. Biol. Med. **16**, 845-850. (10.1016/0891-5849(94)90202-X) 8070690

[130] Kok LDS, Wong YP, Wu TW, Chan HC, Kwok TT, Fung KP. 2000 Morin hydrate a potential antioxidant in minimizing the free-radicals-mediated damage to cardiovascular cells by anti-tumor drugs. Life Sci. **67**, 91-99. (10.1016/s0024-3205(00)00605-6) 10896033

[131] Fang S-H, Hou Y-C, Chang W-C, Hsiu S-L, Lee Chao P-D, Chiang B-L. 2003 Morin sulfates/glucuronides exert anti-inflammatory activity on activated macrophages and decreased the incidence of septic shock. Life Sci. **74**, 743-756. (10.1016/j.lfs.2003.07.017) 14654167

[132] ElGindi M, Sapudom J, Laws P, Garcia-Sabaté A, Daqaq MF, Teo J. 2022 3D microenvironment attenuates simulated microgravity-mediated changes in T cell transcriptome. Cell. Mol. Life Sci. **79**, 508. (10.1007/s00018-022-04531-8) 36063234 PMC11803002

[133] Vorselen D, Roos WH, MacKintosh FC, Wuite GJL, Loon JJWA. 2014 The role of the cytoskeleton in sensing changes in gravity by nonspecialized cells. FASEB J. **28**, 536-547. (10.1096/fj.13-236356) 24249634

[134] Thiel CS et al. 2019 Rapid morphological and cytoskeletal response to microgravity in human primary macrophages. Int. J. Mol. Sci. **20**, E2402. (10.3390/ijms20102402) PMC6567851 31096581

[135] Buken C et al. 2019 Morphological and molecular changes in juvenile normal human fibroblasts exposed to simulated microgravity. Sci. Rep. **9**, 11882. (10.1038/s41598-019-48378-9) 31417174 PMC6695420

[136] Dietz C, Infanger M, Romswinkel A, Strube F, Kraus A. 2019 Apoptosis induction and alteration of cell adherence in human lung cancer cells under simulated microgravity. Int. J. Mol. Sci. **20**, 3601. (10.3390/ijms20143601) 31340547 PMC6678991

